# Rdh54 stabilizes Rad51 at displacement loop intermediates to regulate genetic exchange between chromosomes

**DOI:** 10.1371/journal.pgen.1010412

**Published:** 2022-09-13

**Authors:** Margaret Keymakh, Jennifer Dau, Jingyi Hu, Bryan Ferlez, Michael Lisby, J. Brooks Crickard

**Affiliations:** 1 Deparment of Molecular Biology and Genetics, Cornell University Ithaca, Ithaca, New York, United States of America; 2 Department of Biology, University of Copenhagen, Copenhagen N, Denmark; National Cancer Institute, UNITED STATES

## Abstract

Homologous recombination (HR) is a double-strand break DNA repair pathway that preserves chromosome structure. To repair damaged DNA, HR uses an intact donor DNA sequence located elsewhere in the genome. After the double-strand break is repaired, DNA sequence information can be transferred between donor and recipient DNA molecules through different mechanisms, including DNA crossovers that form between homologous chromosomes. Regulation of DNA sequence transfer is an important step in effectively completing HR and maintaining genome integrity. For example, mitotic exchange of information between homologous chromosomes can result in loss-of-heterozygosity (LOH), and in higher eukaryotes, the development of cancer. The DNA motor protein Rdh54 is a highly conserved DNA translocase that functions during HR. Several existing phenotypes in *rdh54Δ* strains suggest that Rdh54 may regulate effective exchange of DNA during HR. In our current study, we used a combination of biochemical and genetic techniques to dissect the role of Rdh54 on the exchange of genetic information during DNA repair. Our data indicate that *RDH54* regulates DNA strand exchange by stabilizing Rad51 at an early HR intermediate called the displacement loop (D-loop). Rdh54 acts in opposition to Rad51 removal by the DNA motor protein Rad54. Furthermore, we find that expression of a catalytically inactivate allele of Rdh54, *rdh54K318R*, favors non-crossover outcomes. From these results, we propose a model for how Rdh54 may kinetically regulate strand exchange during homologous recombination.

## Author summary

Homologous recombination is an important pathway in repairing DNA double strand breaks. For the purposes of this study, HR can be divided into two stages. The first is a DNA repair stage in which the broken DNA molecule is fixed. In the second stage, information can move from one DNA molecule to another. Enzymes that use the power of ATP hydrolysis to move along dsDNA aid in regulating both stages of HR. In this work we focused on the understudied DNA motor protein Rdh54. We combined genetic and biochemical approaches to show that Rdh54 regulates HR by stabilizing the recombinase protein Rad51 at early HR intermediates.

## Introduction

Homologous recombination (HR) is a universally conserved DNA double-strand break repair (DSBR) pathway that functions to maintain chromosome integrity. Failure in, or mis-regulation of, HR can lead to the loss of genome integrity and the development of human cancers [1,2]. In eukaryotes, HR proceeds through a series of defined steps that begin with the identification of double-strand breaks and the resection of dsDNA into tracts of ssDNA that are bound by replication protein A (RPA). During mitotic DNA repair in eukaryotes, Rad51 replaces RPA on the ssDNA [3]. ssDNA bound by Rad51 is also called recipient DNA, as it will receive information from another DNA molecule during the HR reaction. Rad51 recruits the DNA motor protein Rad54 to promote a systematic search of the genome for a homologous region of DNA [4–9]. Identification of a homologous stretch of DNA results in the formation of a displacement loop (D-loop) intermediate on the donor DNA [10–13].

D-loops are reversible three strand DNA structures that form between donor and recipient DNA molecules. D-loop maturation is regulated by efficiency of DNA base pairing [14], and the activity of specific helicases [10,15,16], topoisomerases [17], and translocases [18,19]. In budding yeast, D-loop maturation is controlled by the highly conserved DNA motor proteins Mph1, Sgs1-Top3-Rmi1 (STR), Srs2, Rdh54, and Rad54. Interactions between these proteins are a major driver in D-loop maturation. For example, Mph1 and STR can disrupt D-loops occupied by Rad54 [17,20], whereas Srs2 cannot [20]. Current models implicate Rad54 in the removal of Rad51 from nascent D-loops leading to D-loop maturation and further strand invasion [19]. DNA synthesis factors PCNA, RFC, and DNA Polymerase delta further stabilize the D-loop [19,21–23] by extending DNA from the 3’ end and restoring the information that was lost with the original dsDNA break.

After DNA extension, D-loops mature through Rad52 mediated annealing of the second end of DNA into an active extended D-loop [24–27], promoting the classical double-strand break repair pathway (DSBR) [13]. Alternatively, extended D-loops can be disrupted and then annealed to the second end of the broken DNA molecule allowing for fill in and completion of DNA repair as part of the synthesis-dependent strand annealing (SDSA) pathway [1,28,29]. If the second end of DNA is not located, the helicase Pif1 can join the DNA replication machinery to create a migrating bubble that can synthesize long tracts of DNA in a process called break induced replication (BIR) [30–32]. When homologous chromosomes instead of sister chromatids are used for DNA repair the net result of these pathways are non-crossover gene conversion (NCO), gene conversion with crossovers (CO), and gene conversion with BIR. The later of these three outcomes can result in the excess transfer of DNA sequence between the recipient and donor DNA molecules. This can lead to allelic loss within the genome, otherwise known as loss-of-heterozygosity (LOH), and is a common cause of certain types of cancers [33].

Rdh54 (Rad54 homolog, a.k.a. Tid1) [34] is a paralog of Rad54 that shares 41% sequence identity [16]. Both proteins are ATP dependent dsDNA translocases and have similar biochemical activities *in vitro*, including DNA supercoiling [35–37], nucleosome remodeling [8,38], enhancement of Rad51 mediated D-loop formation [4,35,36,39,40], and movement along dsDNA [41,42]. Genetic analysis revealed that Rad54 influences inter-sister chromatid and interhomolog recombination [43, 44]. Whereas *rdh54Δ* diploids have a 10-fold reduction in interhomolog gene conversion but had little effect on inter-sister chromatid recombination [45]. However, *rdh54Δ/rad54Δ* diploid strains have enhanced phenotypes over that of individual mutants implying partial redundancy during DNA repair. Whether this is due to direct or indirect effects on HR is unclear. Rdh54 is required to prevent the accumulation of Rad51 on undamaged dsDNA [46,47] and Rad54 can partially compliment this role but has a greater effect on removing Rad51 from sites of DNA damage [46,47]. The model understanding the partial redundancy of these two motor proteins remains under-defined.

There are also several differences between Rdh54 and Rad54. These include Rad54 preferentially initiating Rad51 mediated homology search *in vitro* [4,48]. The stabilization of Rad51 on ssDNA filaments by Rdh54, and that each translocase shares a unique binding site on Rad51 filaments [49]. Major differences in *RDH54* and *RAD54* activity manifests in cells during HR. *RDH54* is also involved the process of inter-chromosomal template switching (ICTS) [16,45]. ICTS events are when D-loops move between chromosomes and are associated with BIR outcomes [50–52]. Both motor proteins are implicated in the process of D-loop expansion and maturation [15,19].

Here we have conducted a study on the biochemical and genetic mechanism by which Rdh54 effects downstream HR outcomes through regulation of D-loop intermediates. We conclude that Rdh54 stabilizes Rad51 at D-loop intermediates by reducing the rate of Rad54 mediated D-loop maturation. The genetic consequence of Rdh54 mediated stabilization is an alteration in the balance between BIR and CO/NCO outcomes. With *RDH54* deletion resulting in an increase in BIR outcomes, and catalytically inactive Rdh54 altering the ratio of CO/NCO outcomes. Finally, we find that Rdh54 phenotypes have stronger association with short tract gene conversion outcomes. From these data we develop a ‘pace car’ model, to explain the regulatory role of Rdh54 in D-loop expansion and its influence on HR outcomes.

## Results

### Deletion of Rdh54 results in an increase in BIR outcomes

Previous reports have used diploid heteroallelic strains to detect rare spontaneous recombination events to define a role for *RDH54* during HR between homologous chromosomes [43–45]. These assays did not differentiate whether *RDH54* was required for direct targeting of the homologous chromosome or may function through a more indirect mechanism. To better understand this question and the role of *RDH54* in HR outcomes between homologous chromosomes, we initiated our studies using a defined genetic reporter that reports on DSB-induced recombination between homologs [53,54]. We selected this reporter for three reasons. First, it would allow us to determine the role of *RDH54* when both sister chromatids are cut [45]. Second, it would allow us to understand how the population of HR outcomes were influenced by *RDH54*. Therefore, even if only a fraction of cells utilized *RDH54* during HR, we would still be able to observe changes to that specific population. Finally, this reporter is well characterized [53–55].

**Fig 1:**
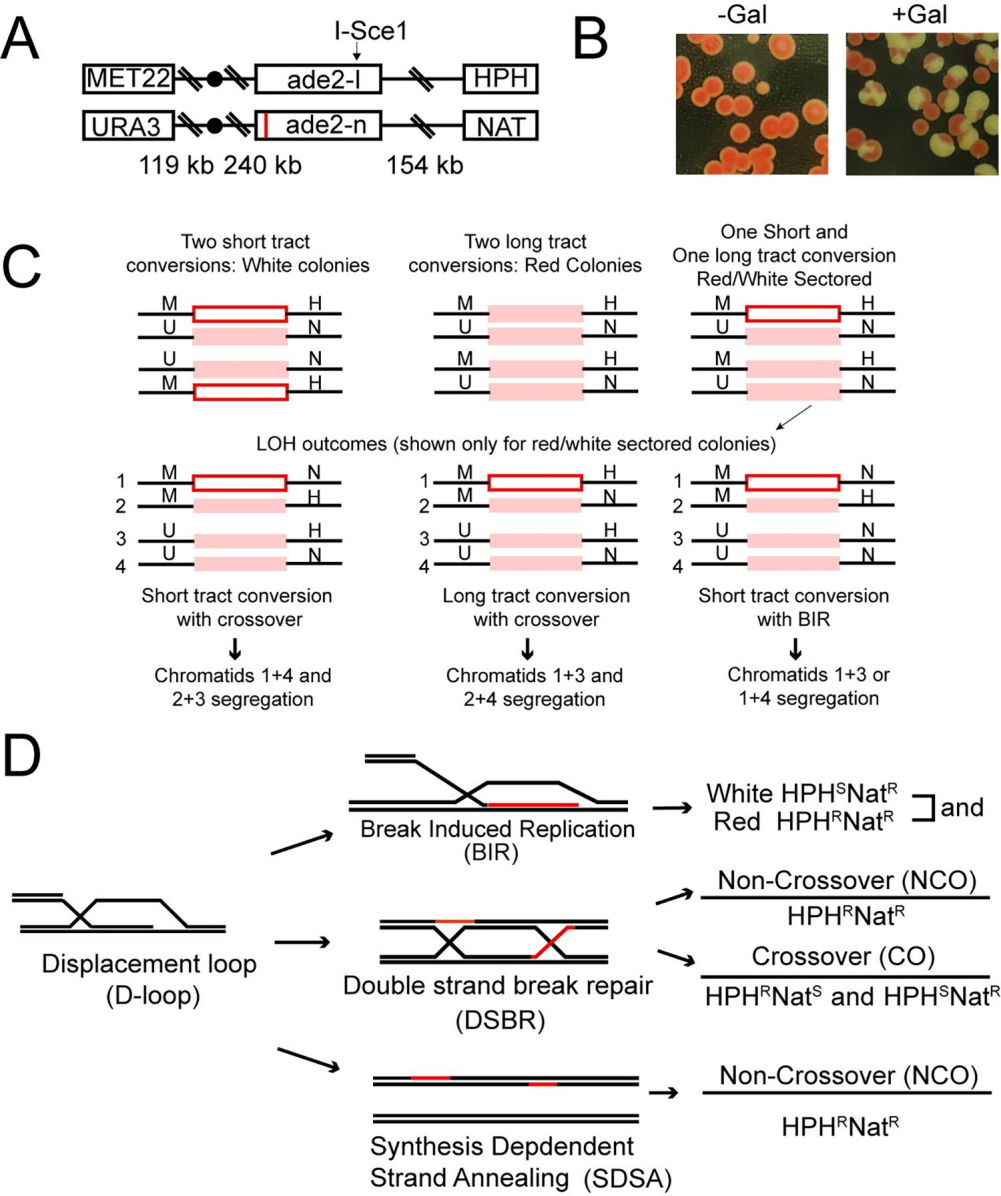
Analysis of RDH54 in post gene conversion recombination. **(A).** Schematic diagram illustrating assay used to monitor recombination between homologous chromosomes during a double strand DNA break. The reporter is located on chromosome XV and only one homolog has an active I-SceI site. The inactivating mutations ade2-n is shown by a red line in the diagram. If Short tract gene conversion occurs (STGC) this mutation is lost and the *ADE2* becomes active. **(B).** Yeast colonies plated after ± induction of I-SceI using galactose. **(C).** Schematic diagram illustrating the potential outcomes for DNA repair via homologous recombination. In the first step gene conversion can occur through long tract or short tract DNA repair. Following cellular division, non-crossover, crossover, and break induced replication (non-reciprocal exchange) outcomes can be determined for each event. **(D).** Cartoon illustration of HR outcomes that can be determined from this assay. The red lines indicate DNA extension.

The reporter relies on an I-SceI nuclease cut site in the ORF of an inactive *ADE2* (*ade2-I*) gene located on chromosome XV (Fig 1A). 154 kbp downstream of the I-SceI cut site is a hygromycin b antibiotic marker (*HPHMX)*. The homologous chromosome contains a non-cleavable inactive allele of *ADE2* (*ade2-n*) that results from a 2 bp deletion in the Nde1 site [56], and a nourseothricin antibiotic marker (*NATMX)* located 154 kbp downstream of *ade2-n* (Fig 1A). The Nde1 is located at the 3’ end of the *ADE2* gene and is 950 bp [56] from the I-Sce1 cut site. Therefore, if long tract gene conversion (LTGC) occurs the repaired homolog will acquire the disrupted gene and the resulting colonies will remain red. However, if gene conversion happens by copying only the 5’ end of the *ADE2* gene, then the repaired copy will be an active *ADE2* gene, and the colonies will turn white. This is short tract gene conversion (STGC). The antibiotic markers are used to determine LOH outcomes due to crossovers or BIR. The differences in the *ADE2* alleles allow for one chromosome to act as a substrate for the I-SceI nuclease, and the homolog to serve as an uncut template for repair. Importantly, to ensure recombination outcomes are not due to loss of chromosomes, *URA3* and *MET22* markers located on the far end of chromosome XV act as markers to report on loss of chromosomes (Fig 1A).

Galactose induced overexpression of I-SceI results in the cleavage of 87–90% of sister chromatids (S2 Table). The cleavage frequency was determined by re-induction assays. DNA repair by gene conversion results in the formation of white colonies (Short tract GC (STGC)), red colonies (Long tract GC (LTGC)), and sectored colonies (Fig 1B and 1C). Sectored colonies are the result of cells with one LTGC, one STGC, and go through the first divisional segregation of chromosomes after plating. Solid red or solid white colonies represent daughter cells that arise from cells in which both sister chromatids have undergone repair by STGC or LTGC. The single color makes the outcomes more difficult to track and interpretation of these colonies more ambiguous (Fig 1C). For our study we have analyzed solid red, solid white, and sectored colonies in separate populations. Within sectored colonies, red sectors rarely retain *ade2-I* and are therefore recombinant outcomes. Occasionally, sectored colonies may grow adjacent to other colonies giving the appearance of colonies with multiple sectors. These colonies were scored carefully when analyzing CO, NCO, and BIR outcomes.

Unselected colonies were replica plated onto selection media to determine different HR outcomes. Crossovers that result in LOH form sectored pairs in which one sector is resistant to hygromycin (HPH) and the other is resistant to nourseothricin (clonNAT), or vice versa. Crossovers without LOH will also form with an equal probability. Therefore, all counted LOH crossovers were multiplied by two. LOH can also result from BIR. Sectored colonies that have gone through BIR will have a white sector that is resistant to clonNAT, but not HPH. This results from the preferential replication of one sister chromatid due to failed second end capture [32,57,58]. Sectored colonies in which both sectors are resistant to HPH and clonNAT are the result of non-crossover DNA repair outcomes (Fig 1C and 1D). For solid red or white colonies BIR outcomes will be sensitive to HPH, but resistant to cloNAT. Colonies that are sensitive to cloNAT, but resistant to HPH are considered BIR like. We did not differentiate these categories and therefore the categories for solid colonies are BIR/BIR like, CO, and NCO. CO in solid-colored colonies were determined by reciprocal exchange of antibiotic sensitivities.

**Fig 2:**
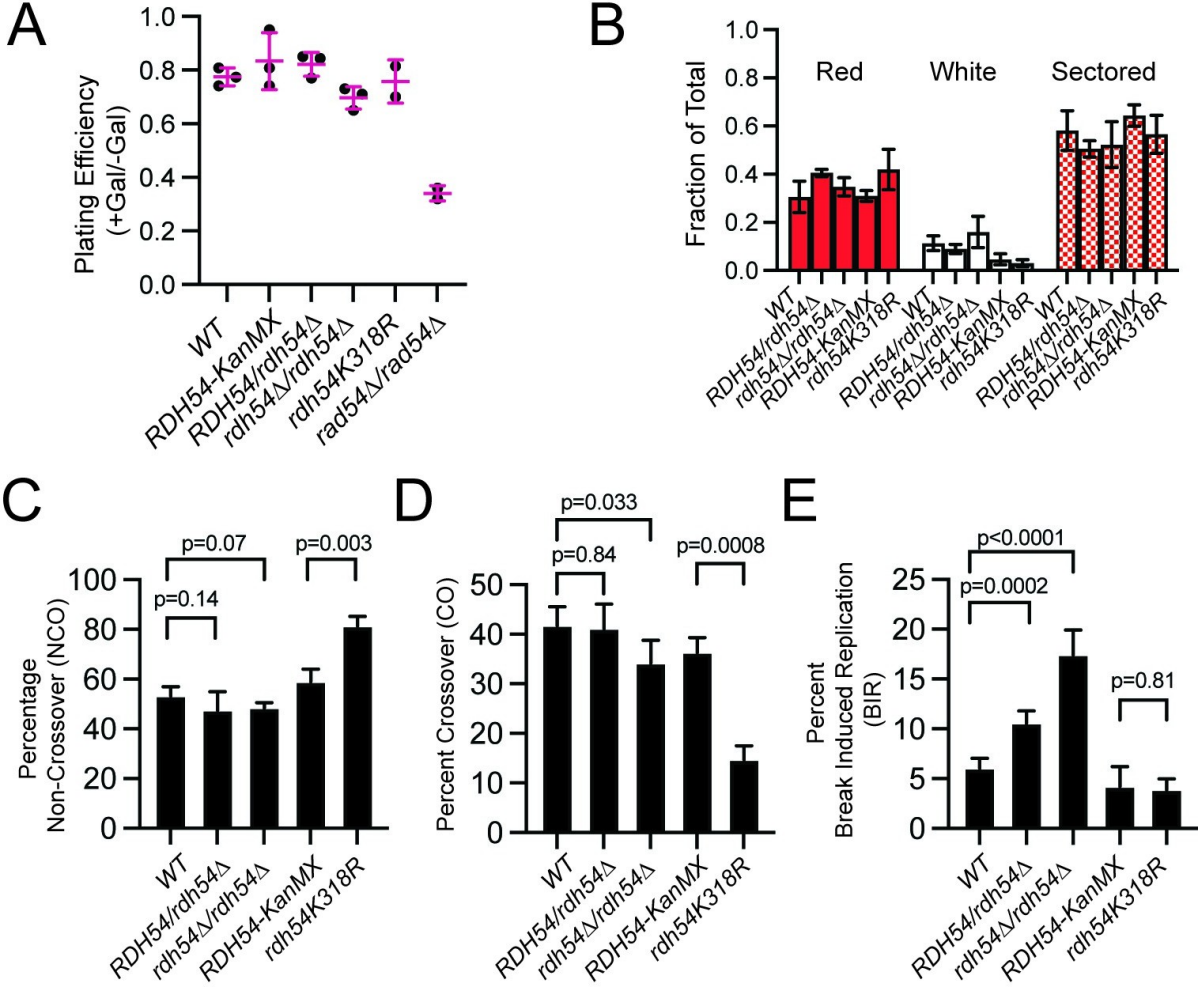
Loss of *RDH54* results in an increase in BIR outcomes. **(A).** Graph representing the plating efficiency of WT, *RDH54-KanMX/RDH54-KanMX*, *RDH54/rdh54*Δ, *rdh54Δ/rdh54Δ*, *rdh54K318R/rdh54K318R*, and *rad54Δ/rad54Δ*. Crossbar and error bars represent the mean and standard deviation of independent measurements. **(B).** Graphical representation of the fraction of cells that are red, white, or sectored after DNA double strand break repair. Strains represented here are *WT*, *RDH54/rdh54Δ*, *rdh54Δ/rdh54Δ*, *RDH54-KanMX/RDH54-KanMX*, *rdh54K318R/rdh54K318R*. The column and the error bars represent the mean and standard deviation of at least three independent experiments. **(C).** Graph representing the percentage of non-crossover outcomes for WT, *RDH54-KanMX/RDH54-KanMX*, *RDH54/rdh54*Δ, *rdh54Δ/rdh54Δ*, *rdh54K318R/rdh54K318R*. Measurements represent the mean and standard deviation of four independent experiments. **(D).** Graph representing the percentage of crossover outcomes for WT, *RDH54-KanMX/RDH54-KanMX*, *RDH54/rdh54*Δ, *rdh54Δ/rdh54Δ*, *rdh54K318R/rdh54K318R*. Measurements represent the mean and standard deviation of four independent experiments. **(E).** Graph representing the percentage of Break induced replication (BIR) outcomes for WT, *RDH54-KanMX/RDH54-KanMX*, *RDH54/rdh54*Δ, *rdh54Δ/rdh54Δ*, *rdh54K318R/rdh54K318R*. Measurements represent the mean and standard deviation of at least four independent experiments.

We first measured plating efficiency after I-SceI cleavage by counting the number of colonies that grew from cultures treated with or without galactose. Reduction in plating efficiency would indicate reduced efficiency in completion of HR. For the WT strain the plating efficiency was 77+/-3.3% (Fig 2A). Similar values were observed for *RDH54/rdh54Δ* (83+/-4.4%), *rdh54Δ/rdh54Δ* (69+/-4.1%), *RDH54-KanMX/RDH54-KanMX* (83.3+/-10.6%), and *rdh54K318R/rdh54K318R* (an ATPase inactive allele of Rdh54) (75.5+/-8%) strains (Fig 2A). In contrast, *rad54Δ/rad54Δ* had a plating efficiency of only 34+/-2.8%, suggesting that cells repaired the I-SceI induced DSB with low efficiency (Fig 2A). Upon reinduction, only one colony (n = 1/410) from the *rad54Δ/rad54Δ* strain was found to be a recombinant (S1 and S2 Tables). This is consistent with previous reports using a similar allelic exchange assay that concluded recombination in *rad54Δ/rad54Δ* cells occurred in only 1% of colonies [59]. It has previously been reported using fluctuation assays that *rad54Δ/rad54Δ* strains result in poor interhomolog recombination and chromosome loss [44,45]. These outcomes would manifest as a low recombination efficiency in our reporter strain, and our results with the *rad54Δ/rad54Δ* strain are consistent with previous observations. In contrast, WT, *RDH54/rdh54Δ*, *rdh54Δ/rdh54Δ*, *RDH54/RDH54-KanMX*, and *rdh54K318R/rdh54K318R* strains all produced red, white, and sectored colonies at similar frequencies upon induction of I-SceI (Fig 2B and S1 Table). Taken together, these data suggest that gene conversion and completion of DNA repair occur with similar efficiency in WT and *rdh54* mutant strains.

We calculated HR outcomes for sectored colonies for each of these strains. The key difference was in the amount of BIR that occurred in *WT*, *RDH54/rdh54Δ*, and *rdh54Δ/rdh54Δ* strains, which contain two, one, or no copies of WT Rdh54, respectively. We observed 6.0+/-1%, of BIR outcomes in the WT strain, 10.5+/-1.2%, in the *RDH54/rdh54Δ* strain, and 17.4+/-2.5%, in the *rdh54Δ/rdh54Δ* strain (Fig 2C–2E and S1 Table). This result suggests that *RDH54* limits BIR in a gene dosage dependent manner. We next complemented the phenotype by integrating WT copies of *RDH54* into the native *RDH54* locus. The integrands had a *KanMX* marker located 125 bp downstream of the end of the *RDH54* ORF. This strain is denoted as *RDH54-KanMX/RDH54-KanMX* and served as a complementing control for *RDH54* mutant alleles. In the *RDH54-KanMX/RDH54-KanMX* strain BIR occurred 4.2+/-2.1% of the time complementing the *rdh54Δ/rdh54Δ* phenotype (Fig 2C–2E and S1 Table). We tested the effect of ATP hydrolysis by Rdh54 on HR outcomes using a catalytically inactive allele of *RDH54* (*rdh54K318R/rdh54K318R)*. Surprisingly, this strain resulted in a distribution of 81.4±3.9%, 14.6+/-2.8%, and 3.8+/-1.2%, NCO, CO, and BIR outcomes, respectively (Fig 2C–2E and S1 Table). We were surprised to see that while *rdh54K318R/rdh54K318R* complemented the BIR phenotype, it also resulted in a significant loss (p = 0.0008) of CO outcomes in favor of an increase in NCO outcomes (p = 0.0036) (Fig 2C and 2D and S1 Table). This observation was unexpected, but consistent with a previous observation made in a haploid yeast strain [15].

To ensure that these outcomes were due to HR and not the loss of chromosomes, sectored colonies were scored for viability on -Ura/-Met plates. Colonies that retain both homologous chromosomes will grow, and if chromosome loss has taken place colonies will fail to grow. No significant increase in chromosome loss was observed for all strains tested (S1 Table). From these data we conclude that *RDH54* has an ATP independent function to reduce BIR and an ATP dependent function to regulate CO/NCO outcomes.

We chose to analyze solid red and white colonies separately from sectored colonies. When analyzing red colonies, the number of non-recombinant events were determined by re-induction assays and then colonies that did not cut were subtracted from the total red cell population. Therefore, recombination outcomes are only evaluated for recombinant colonies. NCO, BIR, and CO outcomes were determined from antibiotic sensitivities after replica plating of un-selected colonies. There were several differences between sectored, solid red, and solid white colonies. In general NCO outcomes were the most substantial part of the solid red and solid white colony outcomes with 89+/-4.8% and 89+/-2.1 of outcomes in the WT strain for red and white respectively (S1 and S3 Figs and S2 and S3 Tables).

BIR outcomes in solid red colonies represented 2.7+/-2.3% of the population. This value increased in both *RDH54/rdh54Δ* (7.1+/-2.8%, p = 0.01) and *rdh54Δ/rdh54Δ* (10.7+/-0.4%, p = 0.02) strains (S1A Fig and S2 Table). The outcome was like that observed for sectored colonies and was complemented by the *rdh54K318R/rdh54K318R* strain (1.7+/-1.4% p = 0.5). CO outcomes were different from those observed in sectored colonies. *RDH54/rdh54Δ* exhibited a slight but insignificant drop in crossover outcomes compared to WT (11.5+/-5.0% versus 7.7+/-2.5%, p = 0.31), and the *rdh54Δ/rdh54Δ* strain had a significant drop in crossovers (3.0+/-2.6%, p = 0.02). Finally, the *rdh54K318R/rdh54K318R* strain also had reduced crossover formation (1.3+/-1.3, p = 0.01) (S1A Fig and S2 Table). From these data we conclude that RDH54 has effects on both BIR and CO outcomes when both sisters are repaired by LTGC.

In solid white colonies *RDH54/rdh54Δ* and *rdh54Δ/rdh54Δ* strains resulted in a significant increase in BIR or BIR like outcomes as compared to WT (1.1+/-1.1% versus 7.4+/-2.9 (p = 0.03) and 17.7+/-6.6% (p = 0.006)). These results were also complemented by the *rdh54K318R/rdh54K318R* (1.4+/-1.4, p = 0.78) strain (S1B Fig and S3 Table). Surprisingly, we observed no CO outcomes for white colonies in the *rdh54Δ/rdh54Δ* (n = 0/33 versus 10/133 (WT)) strain, and a significant drop in CO outcomes in the *RDH54/rdh54Δ* strain (1.0+/-1.5%, p = 0.007). Additionally, the *rdh54K318R/rdh54K318R* strain did not lose CO outcomes entirely but was severely compromised (1.3+/-2.3%, p = 0.01) (S1B Fig and S3 Table). These data suggest that when both sisters are repaired by STGC, COs occur infrequently without *RDH54*, but can occur in an Rdh54 ATP hydrolysis independent manner. We also conclude that consistent with other classes of colonies, BIR or BIR like events increase in a dosage dependent manner in solid white colonies.

### Rdh54 regulates D-loop turnover

**Fig 3:**
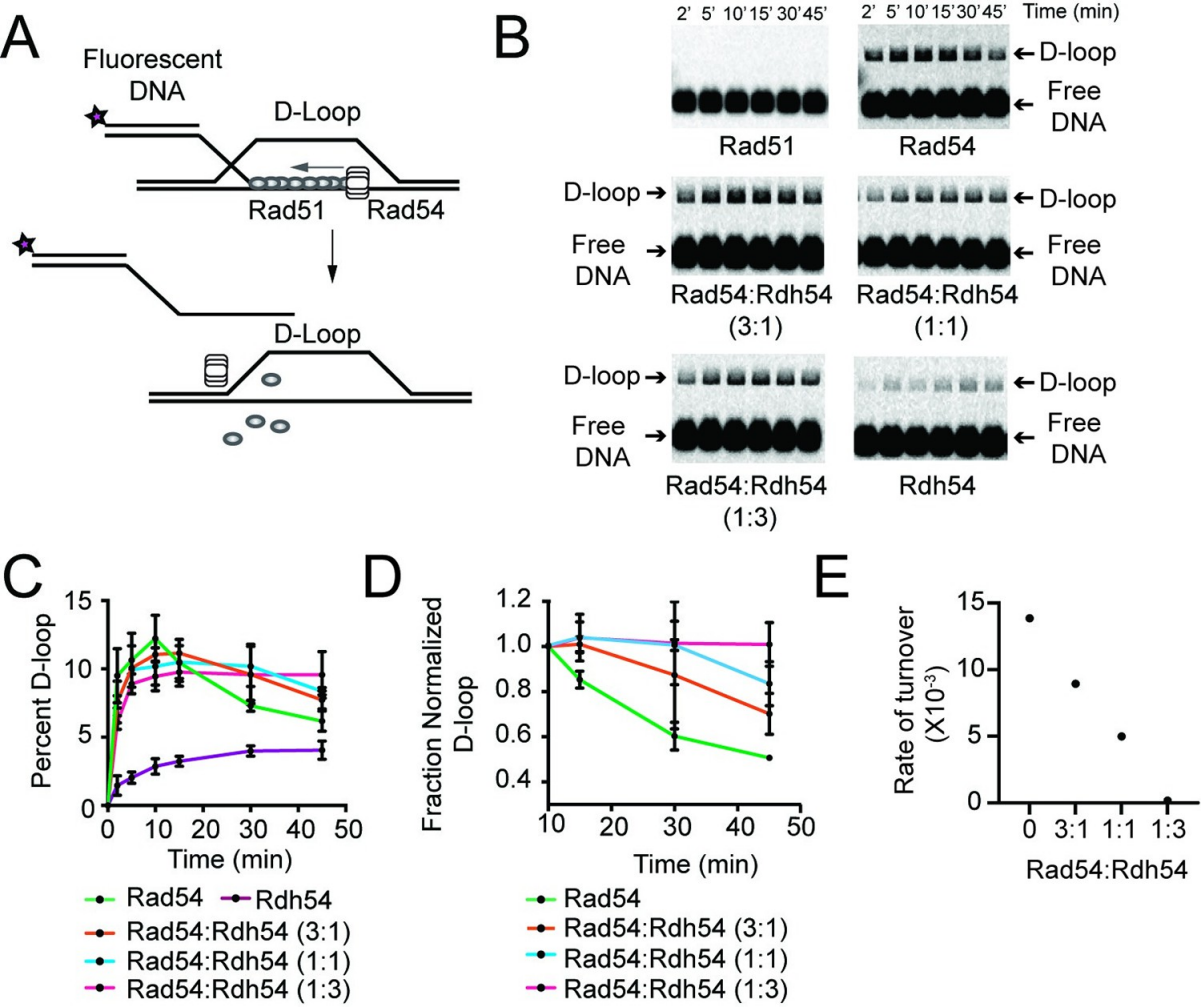
Rdh54 inhibits Rad54 mediated D-loop turnover. **(A).** Cartoon diagram illustrating the experimental design for monitoring Rad54 mediated D-loop maturation. The recipient DNA in this experiment is 21 nt of homologous DNA with 36 bp of non-homologous duplexed DNA **(B).** Representative agarose gels illustrating D-loop turnover for Rad51 alone, Rad51+Rad54 (30 nM), Rad51+Rad54+Rdh54 (10 nM), Rad51+Rad54+Rdh54 (30 nM), Rad51+Rad54+Rdh54 (90 nM), Rad51+Rdh54 (30 nM). **(C).** Quantification of D-loop turnover experiment as a Fraction of D-loop. The error bars represent the standard deviation of three independent experiments. **(D).** Normalized quantification of D-loop turnover. Experiments were normalized by setting the maximum fraction D-loop (10 min) to one and normalizing the other time points to that value. The error bars represent the standard deviation of three independent experiments. **(E).** Graphical depiction of fits of the D-loop turnover phase (10–45 min) of the reaction. The rates are represented from the slope of the best fit line for 0, 3:1, 1:1, and 1:3 molar ratios of Rad54:Rdh54.

Rad54 acts as a motor during HR promoting Rad51 homology search followed by conversion of nascent D-loops into mature D-loops by acting as a dsDNA pump to remove Rad51 [19,21,28]. Previous reports have suggested that Rdh54 competes with Rad54 for D-loop formation. However, these findings were inconclusive in determining whether competition occurred pre- or post-DNA synapsis [60]. Based on our genetic results we hypothesized that post-synaptic inhibition of Rad54 by Rdh54 may support the phenotypes observed in our genetic assay. Therefore, we used an *in vitro* biochemical assay to monitor the formation and maturation of nascent D-loops [19,21]. To effectively monitor both D-loop formation and maturation we used short recipient DNA templates composed of a homologous 21 nt 3’ ssDNA extension from a 35 bp fluorescently labeled (Atto647N) non-homologous duplex region. We used supercoiled pUC19 plasmid as the donor DNA template in these reactions (Fig 3A and S5 Table). We chose to use a short recipient DNA template because the D-loop formation phase can be monitored directly by the appearance of a higher migrating species on a gel. D-loop maturation can then be monitored by disappearance of the shifted product (Fig 3B). The disappearance is caused by Rad54 removal of Rad51 and subsequent instability of the D-loop. We appreciate that the length of DNA used in these assays is much shorter than physiological length [19,60,61]. However, the use of short stretches of homology allows us to address the initial outcomes of the strand invasion reaction, and answer questions that could not be answered using longer lengths of DNA [60]. We state this to make both the limitations and the strengths of our approach clear.

The nascent D-loop is detected by a gel shift in which the recipient DNA migrates higher in the gel due to an interaction with the donor plasmid. This reaction peaks within the first 10 minutes (Fig 3B and 3C). D-loop maturation, as assayed by D-loop disappearance, occurs between the 10-minute and 45-minute time points of the reaction (Fig 3B and 3C). In this assay the addition of Rad51 alone is not sufficient for D-loop formation [62]. The addition of Rad54 together with Rad51 initially catalyzes D-loop formation and promotes D-loop disappearance (Fig 3B and 3C). We monitored reactions at 2, 5, 10, 15, 30, and 45 min. As expected, D-loops formed in the first 10 minutes of the reaction and then began to disappear between 10 minutes and 45 minutes, with 50% of the maximum number of D-loops that formed lost after 45 min (Fig 3B and 3C).

We titrated Rdh54 against a fixed concentration of Rad51, Rad54, and RPA creating molar ratios of 1:3, 1:1, and 3:1 Rad54:Rdh54. Rdh54 in combination with Rad54 had little effect on the formation of D-loops with only a modest reduction in D-loop formation when Rdh54 was present at 3-fold molar excess relative to Rad54 (Fig 3B and 3C). Surprisingly, a reduction in D-loop maturation was observed, with the fraction of D-loop disappearance changing from 0.5 +/- 0.1, 0.7+/-0.1, 0.83+/- 0.1, and 1.0+/-0.1 at 0, 3:1, 1:1, 1:3 molar ratios of Rad54:Rdh54, respectively. This represented a 29%, 39%, and 100% reduction in D-loop maturation (Fig 3C–3E). We also measured the rate of D-loop dissociation and found that the rates of decay were 13.8 x 10^−3^ min^-1^, 8 x 10^−3^ min^-1^, 4.9 x10^-3^ min^-1^, and 0.18 x 10^−3^ min^-1^ at 0, 3:1, 1:1, 1:3 molar ratios of Rad54:Rdh54, respectively (Fig 3E). Rdh54 in combination with only Rad51 was able to catalyze D-loop formation but did not promote D-loop dissociation within our observation window.

To determine if inhibition was due to the ability of Rdh54 to inhibit the ATPase activity of Rad54, we mixed Rdh54K318R with Rad54 in equal molar amounts in the presence or absence of Rad51. We observed that there was no change in the Rad54 ATPase activity in either case, suggesting the ATPase of Rad54 was unaffected (S2A Fig). In an effort to determine if our ratios of Rad54:Rdh54 fell within a physiological range, we measured the total number of Rad54 and Rdh54 molecules in the nucleus [63]. Our measurements focused on the ratio of Rad54 and Rdh54 in diploid cells. We found that Rad54 occurred at a mean 430 molecules per nucleus and Rdh54 occurred at 673 molecules per nucleus (S2B Fig). This gives a predicted ratio of Rdh54:Rad54 of approximately 1.6:1, suggesting that our *in vitro* experiments were performed around the physiological ratio of the two translocases. Together these data suggest that Rdh54 can reduce D-loop maturation under physiological protein concentrations.

### Rdh54 reduces the rate of D-loop maturation

**Fig 4:**
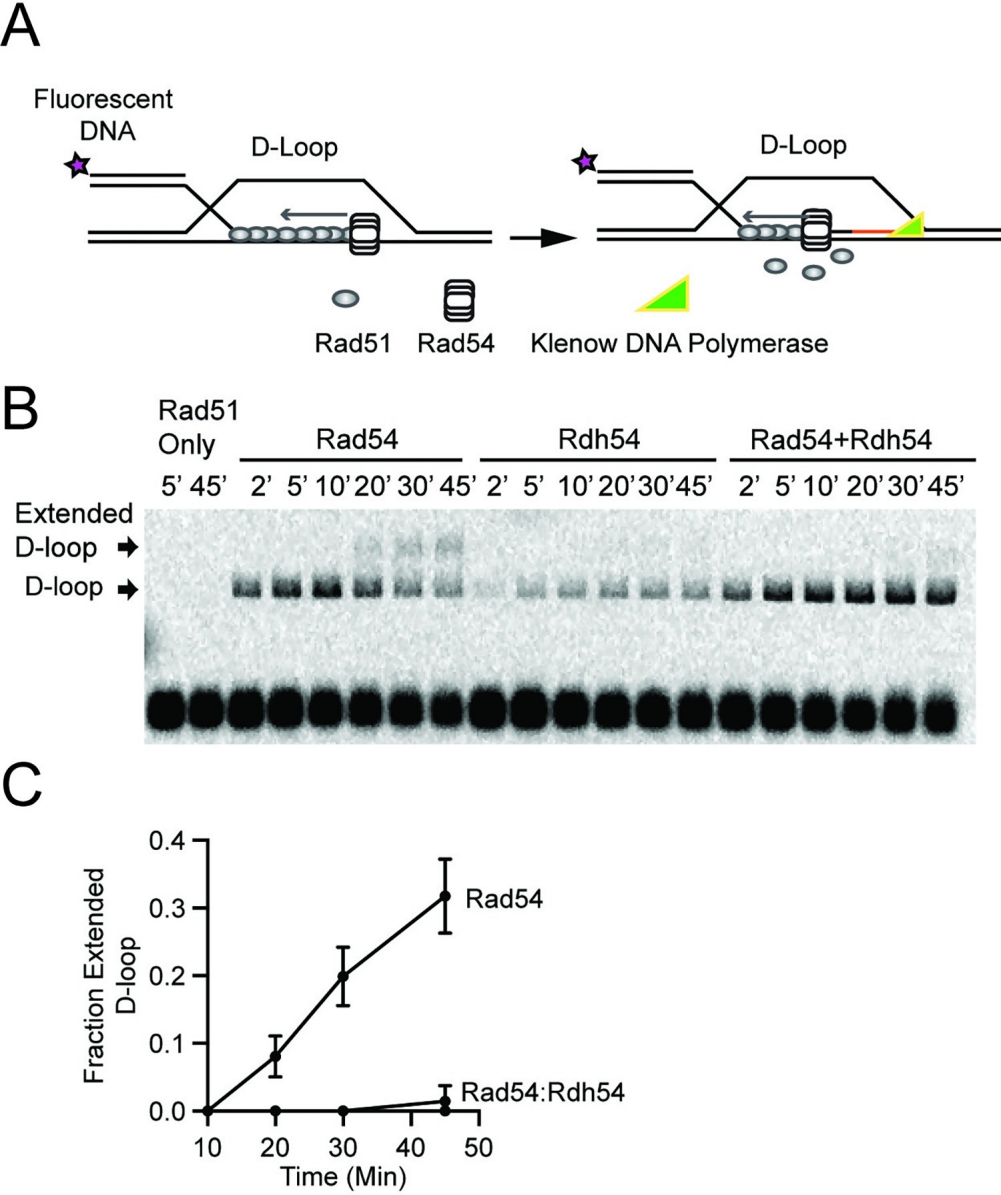
Rdh54 prevents Rad54 from creating accessible 3’ end at D-loop intermediates. **(A).** Cartoon diagram illustrating the experiment to monitor the 3’ end accessibility by using Klenow (exo-) to extend the nascent D-loop. The recipient DNA used in this experiment was 21 nt of homologous ssDNA and 36 bp of duplexed non-homologous DNA **(B).** Representative gel illustrating D-loop extension for Rad51 alone, Rad51+Rad54 (30 nM), Rad51+Rdh54 (30 nM), and Rad51+Rad54 (30 nM) +Rdh54 (30 nM). **(C).** Quantification of extended D-loop fraction. The error bars represent the standard deviation for three independent experiments.

As Rad54 promotes D-loop maturation the 3’ end of the nascent D-loop becomes accessible to the DNA replication machinery. We modelled this D-loop maturation process *in vitro* using Klenow fragment (exo-) as a DNA polymerase to form extended D-loops (Fig 4A). We hypothesized that Rdh54 could limit the accessibility of the 3’ end of newly formed D-loop intermediates by reducing D-loop maturation [21]. The formation of extended D-loops was monitored by a shift in the size of the D-loop within the gel (Fig 4B). No D-loops or shifted products formed in the absence of Rad54 (Fig 4B). When Rad54 was present, extended D-loops represented 0, 8.0.1+/-3.0%, 19.8+/-4.3%, 31.7+/-5.4% of D-loop populations at 10, 20, 30, and 45 minutes, respectively (Fig 4B and 4C). In contrast, the addition of Rdh54 resulted in the formation of D-loops but did not yield extension products in the time window of the experiment (Fig 4B and 4C). When Rad54 and Rdh54 were mixed, the formation of extended D-loops was inhibited almost completely, with only a small fraction of extended D-loops (1.4+/-2.4%) detected at the 45 min time point (Fig 4B and 4C). Together with the result that Rdh54 limits Rad54 mediated D-loop maturation, we are confident that these data suggest Rdh54 is limiting Rad54 activity at D-loops.

**Fig 5:**
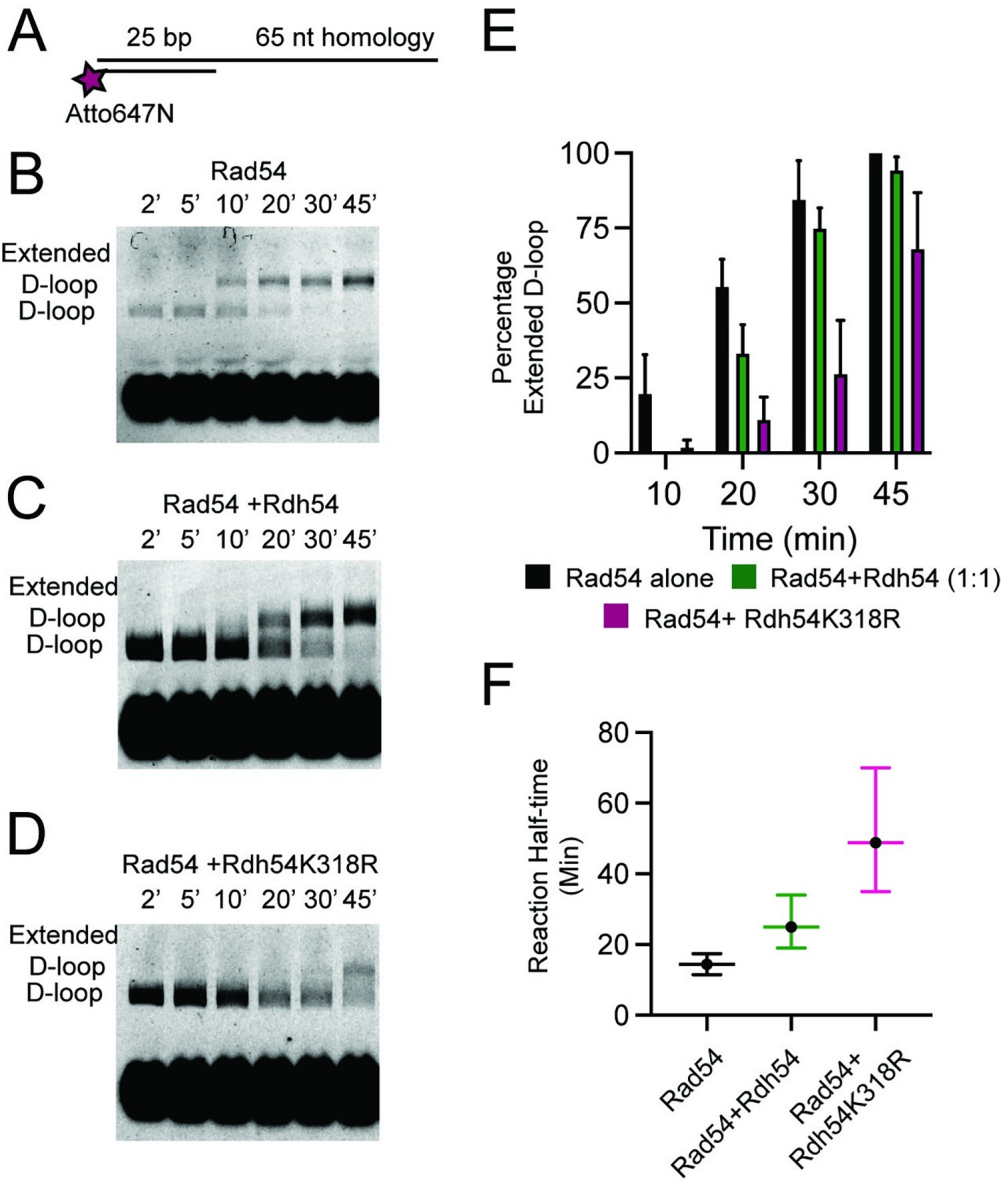
Rdh54 kinetically effects D-loop maturation on longer DNA templates. **(A).** Schematic diagram illustrating the longer fluorescently recipient DNA used in this experiments. The recipient DNA used in this experiment was 65 nt of homologous ssDNA and 25 bp of non-homologous dsDNA **(B).** Representative agarose gel representing a D-loop extension assay with Rad54 (30 nM) at 2, 5, 10, 20, 30, and 45 minutes. **(C).** Representative agarose gel representing D-loop extension assay with Rad54 (30 nM) + Rdh54 (30 nM) at 2, 5, 10, 20, 30, and 45 minutes. **(D).** Representative agarose gel representing D-loop extension assay with Rad54 (30 nM) + Rdh54K318R (30 nM) at 2, 5, 10, 20, 30, and 45 minutes. **(E).** Bar graph representing quantification of the fraction of D-loops that are extended at 10, 20, 30, and 45 min. The error bars represent the mean and standard deviation of 4–10 independent experiments. **(F).** A plot illustrating the reaction half-time generated from a single-phase association fit of the percentage D-loop extension. The crossbars represent the mean, and the error bars represent the upper and lower limit of the half-time based on the fit of the data.

Even though our goal was to test the outcomes of initial strand invasion events when both Rad54 and Rdh54 are present, the small size of our recipient DNA raised the question whether a longer length of DNA would have similar outcomes. To test this, we used a recipient DNA probe with 65 nt of homologous ssDNA and 25 bp of non-homologous dsDNA (Fig 5A and S5 Table). We measured the extension of the 3’ end of newly paired DNA. Anecdotally, the 3’ end extension was more efficient for 65 nt of homology than for the 21 nt of homology. With 19%+/-12%, 55%+/-9%, 84+/-13%, and 100% of the D-loops extended at 10, 20, 30, and 45 minutes respectively. When Rdh54 was mixed in equal amounts with Rad54, the extension of the 3’ end was slower than Rad54 alone. With 0, 33+/-9%, 74+/-6%, and 94+4% D-loops extended at 10, 20, 30, and 45 min respectively (Fig 5B, 5C and 5E). To measure the differences in the reaction, we fit these data to a single-phase association curve that was bounded at 0 and 100%. From this fit we calculated a reaction half-time for Rad54 alone of 14.5+/-3 minutes, and for Rad54+Rdh54 of 25+/-7.5 minutes (Fig 5F). From this data we conclude that on longer stretches of homology Rdh54 only slows the rate of D-loop maturation and does not completely inhibit D-loop expansion.

Because longer stretches of DNA homology revealed the kinetic nature of Rdh54 mediated inhibition, we next measured the effect of the catalytically inactive Rdh54K318R. Surprisingly, we found that Rdh54K318R had greater impact on the rate of 3’ end accessibility than WT Rdh54. With 1+/-2.5, 11+/-7.5, 26+/-17, and 68+/-18% D-loop extended at 10, 20, 30, and 45 minutes respectively. The half-time of the reaction was 48 +/-17 minutes (Fig 5D, 5E and 5F). These data indicate a role for the ATPase of Rdh54 in D-loop maturation. From these data we conclude that Rdh54 kinetically regulates D-loop maturation, and that the ATPase is involved in this reaction.

### The activity of Rdh54 at D-loops is affected by binding position

Rad54 and Rdh54 bind to Rad51 filaments at unique sites [48,64–66]. Unique binding sites were discovered by learning that the meiosis specific protein Hed1 inhibited Rad54 binding to Rad51 but did not affect Rdh54 binding to Rad51 [65]. This led to the development of chimeric versions of Rad54 and Rdh54 in which the N-terminal domains of these two proteins were switched. Thus, placing the Rad51 binding domain of Rad54 on the ATPase of Rdh54, and vice versa. These chimeric proteins are aa 1–260 of Rad54 fused to aa 260–958 of Rdh54 for ^Rad54N^Rdh54, and aa 1–280 from Rdh54 fused to aa 281–898 from Rad54 for ^Rdh54N^Rad54. Important biochemical features of these chimera include, that both ^Rad54N^Rdh54 and ^Rdh54N^Rad54 are ATPase active and reflect the activity of their parent ATPase. For example, ^Rdh54N^Rad54 has comparable ATPase activity to Rad54, but has a severe phenotype in cells [48]. Here we have used the chimeric mutants ^Rad54N^Rdh54 and ^Rdh54N^Rad54 to monitor the effect of translocase positioning on D-loop maturation (S3A Fig). As has been previously reported, the D-loop formation phase of ^Rad54N^Rdh54 was comparable to that of WT-Rdh54, with peak D-loop formation of 5.2±1.2% and 4.8+/-0.2%, respectively (S3B and S3C Fig). Also consistent with previous work, the ^Rdh54N^Rad54 hybrid protein was defective in D-loop formation, with peak D-loop formation of 3.1+/-0.2% as compared to 8.0+/-1.8% for the WT Rad54 (S3B and S3C Fig). It should be noted that a kinetic delay precedes peak D-loop formation with ^Rdh54N^Rad54. This is due to defects in homology search [48].

The D-loop maturation phase (10–45 min) of the reaction for both ^Rad54N^Rdh54 and ^Rdh54N^Rad54 indicate defective D-loop dissociation, with 91% and 90% of D-loops remaining after 45 minutes, respectively. For WT-Rdh54, 114% of D-loop signal remains after 45 minutes (S3B and S3C Fig). It should be noted that we did not carry the reactions on for an extended time. This makes direct comparison of the D-loop dissociation phase of the reaction difficult because D-loops do not form at equivalent rates in each case. However, if Rdh54, ^Rad54N^Rdh54, or ^Rdh54N^Rad54 trigger D-loop maturation it occurs on a time scale that is well beyond the time frame of this experiment and may not be sufficient to promote effects *in vivo*. In fact, ^Rdh54N^Rad54 fails to complement *rad54Δ* phenotypes [48]. Whether this is due to a failure during homology search or D-loop maturation remains unclear as ^Rdh54N^Rad54 is deficient for both activities *in vitro*.

**Fig 6:**
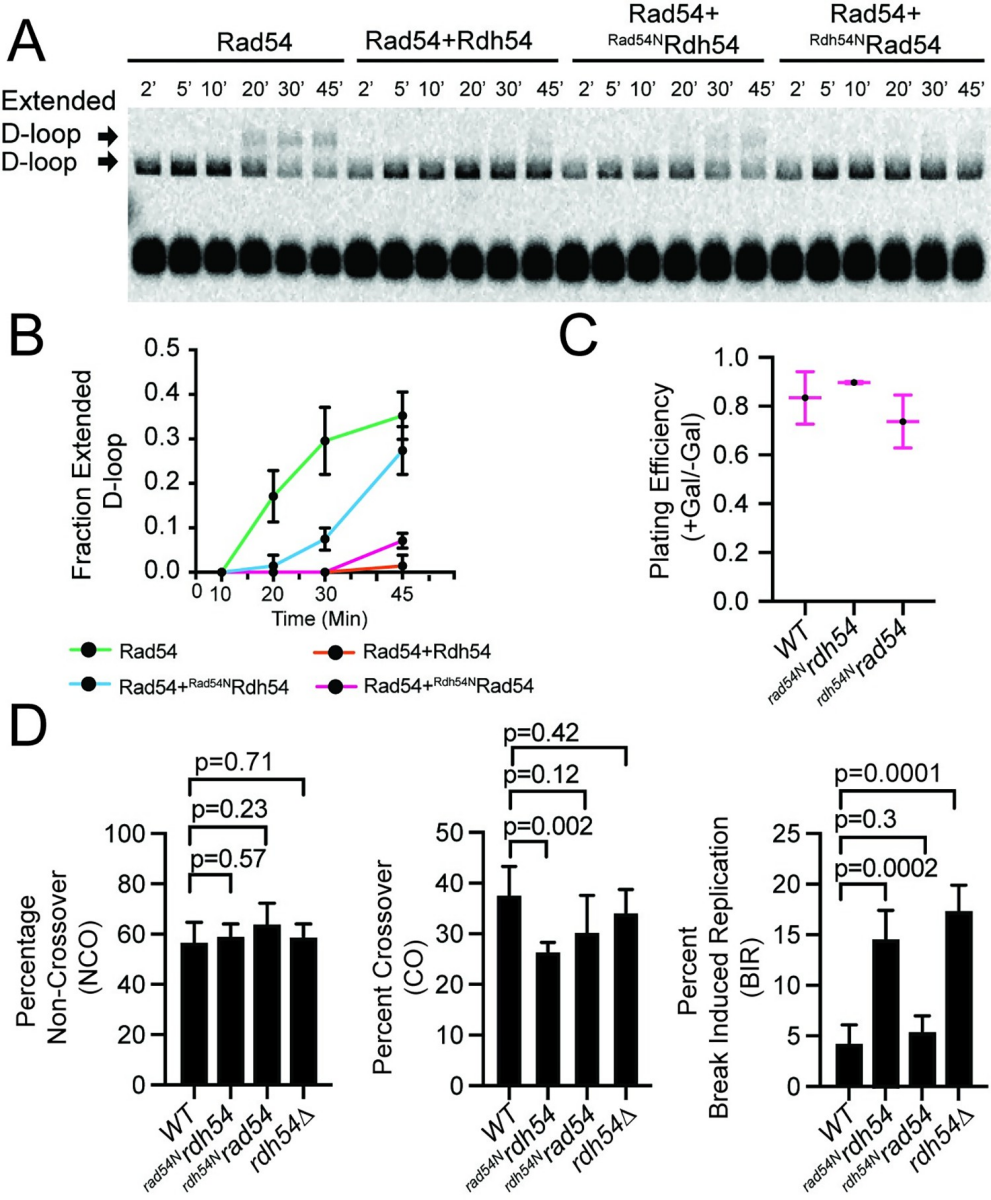
Rad51 binding site switching of Rdh54 and Rad54 alters 3’ end accessibility and post gene conversion outcomes. **(A).** Representative gel image of experiments designed to test how efficiently Rdh54, ^Rad54N^Rdh54, and ^Rdh54N^Rad54 interfere with Rad54 mediated 3’ end accessibility to Klenow fragment. The recipient DNA in this experiment was 21 nt of homologous ssDNA and 36 bp of non-homologous DNA **(B).** Quantification of extended D-loops for samples with Rad54, Rdh54, ^Rad54N^Rdh54, and ^Rdh54N^Rad54. The data points and error bars represent the mean and standard deviation of at least three independent experiments. **(C).** Graph representing the plating efficiency (+Gal/-Gal) for WT-*KanMX*, ^*rad54n*^*rdh54*, and ^*rdh54n*^*Rad54* yeast strains. The data represents the mean and standard deviation for four independent experiments. **(D).** Bar graphs representing the NCO (left), CO (middle), and BIR (right) outcomes for *WT-KanMX*, ^*rad54n*^*rdh54*, and ^*rdh54n*^*rad54*. The data represent the mean and standard deviation of at least four independent experiments. The *WT* and *rdh54Δ* results are reproduced from previous figures for comparison purposes.

After determining that the chimeric mutants of Rad54 and Rdh54 were unable to promote D-loop dissociation in the observation window of the experiment, we next asked whether these mutants could inhibit Rad54 mediated D-loop maturation. To provide the most efficient assay to test this, we used recipient DNA of 21 nt in length. Our expectation from this experiment was that if the position of Rdh54 binding was important for reducing Rad54 mediated D-loop maturation, then the ^Rad54N^Rdh54 mutant would fail to prevent 3’ end extension, and D-loop maturation. In contrast, the ^Rdh54N^Rad54 mutant would prevent the extension of the 3’ end. Consistent with this prediction, we observed that ^Rdh54N^Rad54 severely inhibited extended D-loop formation with 0%, 0%, 0%, and 1.4+/-1.2% extended D-loops observed at 10, 20, 30, and 45 min, respectively (Fig 6A and 6B). The ^Rad54N^Rdh54 was unable to prevent D-loop maturation, with 0, 1.4+/-1.2%, 7.4+/-2.5%, and 27.0+/-5.0% of extended D-loops observed at 10, 20, 30, and 45 min, respectively (Fig 6A and 6B). We believe the slight reduction in the extended D-loops formed by ^Rad54N^Rdh54 relative to WT Rad54 was due to competition for the same binding site on Rad51. From this experiment we conclude that the position of Rdh54 binding to Rad51 regulates Rad54 mediated D-loop maturation.

To genetically test our hypothesis, we integrated the ^*rdh54N*^*rad54* and ^*rad54N*^*rdh54* alleles into the *RDH54* gene in our reporter strain, creating diploid strains with these mutant alleles. We hypothesized that the ^*rdh54N*^*rad54* mutant would prevent the onset of BIR but would be unable to allow crossovers to occur, and that the ^*rad54N*^*rdh54* mutant would be unable to prevent BIR outcomes but would be able to promote crossover formation. As with other *RDH54* alleles, there was no significant impact on gene conversion (S4 Fig). Cells did not have reduced recombination efficiency upon cleavage by I-SceI (Fig 6C). For ^*rad54N*^*rdh54* allele in sectored colonies we observed 26.4+/-2.2%, 61.0+/-4.2%, 14.8+/-2.8% of CO, NCO, and BIR outcomes, respectively (Fig 6D). In contrast, for the ^*rdh54N*^*rad54* allele in sectored colonies we observed 30.3+/-7.2%, 64.1+/-8.0%, 5.5+/-1.5% of CO, NCO, and BIR, respectively (Fig 6D and S1 Table). These data indicate that the ^*rad54N*^*rdh54* allele failed to complement the BIR phenotype. As with the other strains analyzed here, there was not a significant loss of chromosomes for ^*rad54N*^*rdh54* or ^*rdh54*^*rad54* (S1 Table).

We measured outcomes for individual red and white colonies. The major findings were like those observed for sectored colonies with several exceptions. For red colonies, ^*rad54N*^*rdh54* resulted in increased BIR/BIR like events (7.5+/-3.0%, p = 0.07) (S5A Fig and S2 Table). In ^*rad54N*^*rdh54* mutants, white colonies resulted in BIR/BIR like outcomes 30+/-9% (S5B Fig and S3 Table) of the time. This stood in stark contrast to WT and ^*rdh54N*^*Rad54* strains in which BIR occurred in only 1.5+/-2.5% and 1.9+/-0.3% of outcomes respectively (S5B Fig and S3 Table). In contrast to the *rdh54Δ* strain, there was no reduction in CO outcomes in solid red colonies for ^rad54N^Rdh54 (8.5+/-1.8%, p = 0.31) or ^rdh54N^Rad54 (9.1+/-2.0%, p = 0.49) strains (S5A Fig). In solid white colonies no CO were observed for the ^*rad54N*^*rdh54* strain (n = 0/78, p = 0.0002) (S5B Fig and S3 Table). These results are surprising, but consistent with the *rdh54Δ* strain. This mutant phenotype was not observed for the ^*rdh54N*^*rad54* strain (8.8+/-3.1%, p = 0.6).

For BIR/BIR like outcomes and CO outcomes we quantified frequency relative to WT for the sectored, solid red, and solid white categories. All BIR/BIR like categories occurred at a frequency relative to WT greater than 1 (S6A Fig). For *rdh54Δ* and ^*rad54N*^*rdh54* strains in solid white colonies the frequency relative to WT was 17 and 30 times greater respectively in BIR or BIR like outcomes. This stands in contrast to red and sectored colonies in which BIR events occurred at only a 4 times greater frequency than WT in the *rdh54Δ/rdh54Δ* strain (red and sectored). For the ^*rad54N*^*Rdh54* strain a 2.7 (red) and 3.4 (sectored) increased frequency was observed. By comparison the ^Rdh54N^Rad54 strain produced a 1.0-, 1.6-, and 1.26 increase in frequency for solid white, solid red, and sectored colonies respectively (S6A Fig). These data suggest that solid white colonies show the strongest *rdh54* mutant phenotypes.

We performed a similar analysis for CO outcomes. Almost all values will be less than 1 because in many cases CO are reduced (S6B Fig). For example, the frequency relative to WT for the *rdh54Δ/rdh54Δ* strain was 0, 0.27 and 0.91 for white, red, and sectored respectively. For the *rdh54K318R* strain the relative frequency was 0.18, 0.12, and 0.37 for white, red, and sectored respectively. Finally, we observed frequencies of 0, 0.72, and 0.70 for ^*rad54n*^*rdh54* strain in white, red, and sectored colonies respectively (S6B Fig). Together this analysis suggests that the effects of *RDH54* on CO are greater on homotypic sister repair. Overall, these data suggest that *RDH54* may have greater influence over cells during STGC, and that where Rdh54 binds to Rad51 is important for this activity.

## Discussion

Our study provides new insight into the role of Rdh54 during homologous recombination. The data highlights the ability of Rdh54 to act as a soft barrier during D-loop maturation. Biologically, the consequence of this activity is to prevent the onset of BIR in an ATP independent manner. The loss of Rdh54 ATP hydrolysis results in an increase in NCO outcomes at the expense of CO outcomes. Finally, we observed that Rdh54 was required for CO formation in cells in which both sister chromatids had undergone STGC, and that this activity was only partially dependent on ATP. Together, our data supports a model where Rdh54 stabilizes Rad51 at D-loops and regulates the maturation of HR intermediates.

### Genetic consequences of *RDH54* activity

Previous reports have implicated *RDH54* in interhomolog gene conversion [44,45]. Both studies used a fluctuation assay to measure spontaneous recombination between *leu2* heteroalleles. The fluctuation test results from random DSBs in the DNA which can occur through replication stress or spontaneous recombination during G1 of the cell cycle [67,68]. The red/white reporter reduces chromosome choice by cutting both copies of the sister chromatid forcing DSB repair through the homologous chromosomes. Using this assay, we observed phenotypes that reflected a dosage dependent increase in BIR outcomes and gene conversion tract length dependent transitions to CO and BIR outcomes.

There are several possible reasons for our observations. The simplest explanation to our data is that delaying Rad54 mediated removal of Rad51 during D-loop maturation may directly influence BIR outcomes. Recently, evidence has emerged that ensuring kinetic balance of the two ends of a double strand break is responsible for competition between the BIR and SDSA pathways [32]. Our results are similar to those observed for the *rad52R70A* annealase deficient mutant [32]. It is possible that reducing the rate of D-loop maturation may contribute to achieving kinetic balance between the two ends of a DSB. Thus, increases in BIR result when Rdh54 cannot stabilize Rad51.

Alternatively, *RDH54* is involved in ICTS, but not in initiation of short-range BIR events [50]. This leads to an alternative possible interpretation of our data. In our assay the I-Sce1 site used to trigger a DSB is located 154 kbp away from the antibiotic marker. To register as a BIR event then 154 kbp of DNA must be copied. If the active BIR complex were to switch templates between the cut site and the marker, then LOH by BIR would not be observed. In WT cells when Rdh54 is present template switching may be prevalent [69], and this may be the case. In the absence of Rdh54 or when Rdh54 binds at a different location on Rad51, then template switching does not occur and an increase in LOH by BIR is observed through long range replication. As discussed below, this may be a consequence of Rad51 stabilization resulting in persistent strand exchange activity.

### Rdh54 regulates Rad51 D-loop dynamics

Mechanistically, Rad54 and Rdh54 promote removal of Rad51 through a pump like mechanisms that induces Rad51 ATP hydrolysis as they translocate along dsDNA [19,37]. Our current findings suggest that Rdh54 can kinetically regulate the activity of Rad54 at nascent D-loop intermediates, leading to a reduced rate of D-loop maturation. Previous reports have shown that Rdh54 limits D-loop size and reduces the formation of long D-loop products [60]. These experiments did not differentiate between a model where Rdh54 functioned as a roadblock to Rad54 post strand invasion, or if Rdh54 competed with Rad54 to initiate strand invasion. In our experiments we use significantly shorter recipient DNA fragments (21 and 65 versus 600). While these fragments may not be of physiological length, they do provide insight into what is happening during initial strand invasion events. We did not observe inhibition of nascent D-loop formation, but instead observed inhibition of D-loop maturation through our analysis of DNA 3’ end accessibility. These data support the second model where Rdh54 does not compete with Rad54 for D-loop formation, but instead regulates D-loop expansion by providing a barrier to Rad54 translocase activity. While the exact mechanism by which Rdh54 prevents Rad54 activity is unclear, a suitable hypothesis is that Rdh54 antagonizes Rad54 induced ATP hydrolysis by Rad51.

Others have observed that in *rdh54Δ* strains there is an increase in D-loop formation in a haploid specific manner [60]. This would seem counterintuitive to our model. However, our biochemical data predict that short nascent D-loops will undergo conversion to mature D-loops only in the absence of Rdh54, or over extended time frames. The increase in D-loops may reflect short strand invasion products that mature in the absence of Rdh54 that do not when it is present. By inhibiting the removal of Rad51 and subsequent maturation of strand invasion, D-loops may be more dynamic and capable of facilitating re-invasion or template switches. Our reasoning is that stable Rad51 filaments are likely to remain active even after initial identification of a homologous sequence and will remain active until fully removed by Rad54.

We observed that the *rdh54K318R* allele resulted in an increase in NCO and a reduction in CO outcomes. Biochemically Rdh54K318R allowed D-loop maturation at a lower rate than WT- Rdh54. These observations are consistent with recent findings that a haploid *rdh54K318R* strain had reduced crossover frequency, as well as a kinetic delay in general repair in haploid yeast [15]. One interpretation of these findings is that Rdh54 must move or translocate away from sites of recombination for crossovers to occur. Thus, acting as a potential switch to regulate crossover/non-crossover outcomes. How and when Rdh54 translocates away from the D-loop remains unclear.

### Implications of D-loop dynamics and partial redundancy

**Fig 7:**
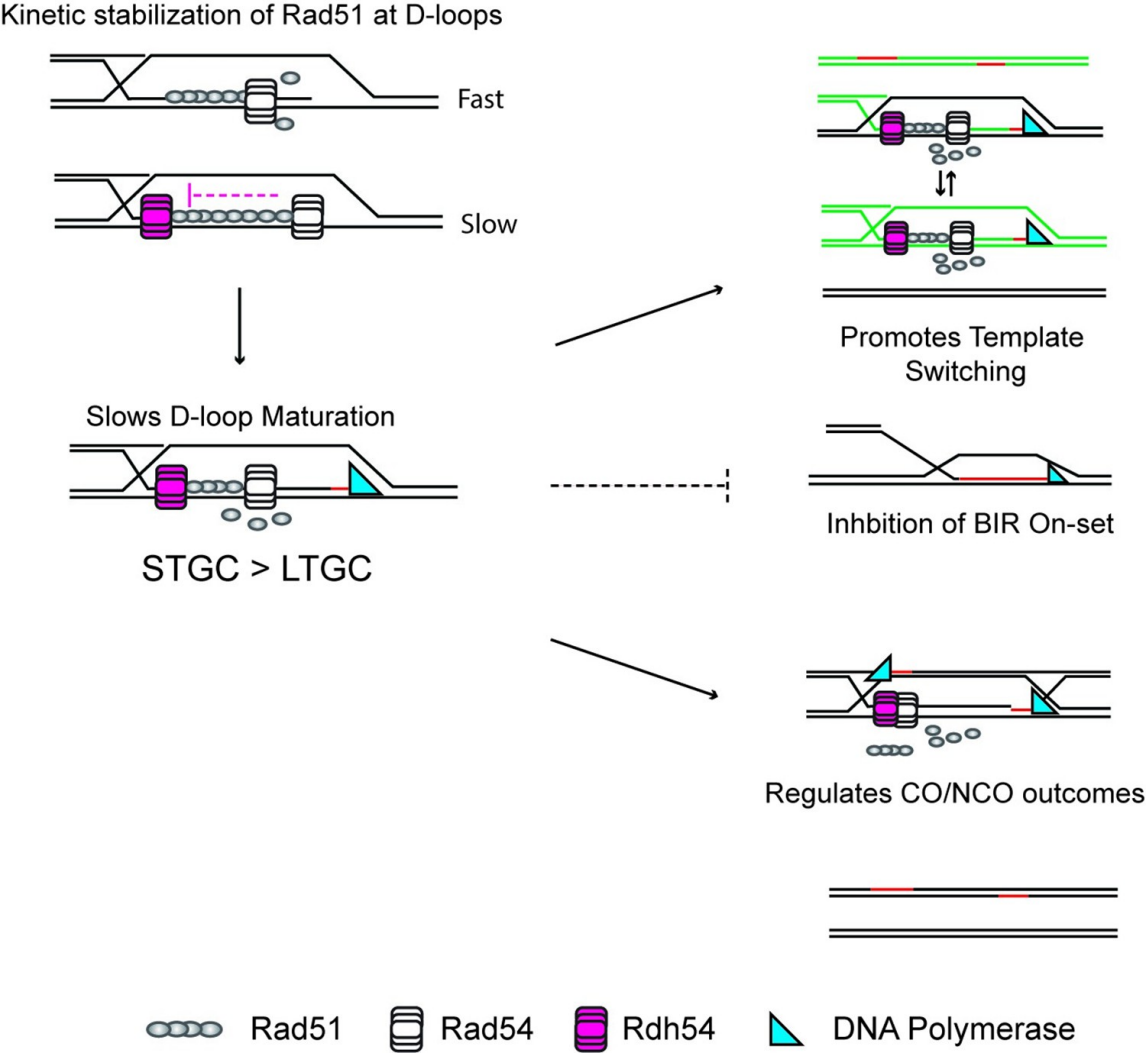
Model for how Rdh54 alters HR pathway choice. A cartoon diagram illustrating clearance of Rad51 when Rdh54 is present. Rdh54 slows the rate of Rad51 clearance leading to an increase in potential template switching, or a decrease in BIR initiation. Rdh54 translocation can then regulate NCO/CO outcomes through movement away from the D-loop. The effect on transitions from GC are greater for STGC than LTGC. The second step should also be considered possible through SDSA. Note exact position of Rdh54 relative to Rad54 in the D-loop is currently unknown, and our positioning is speculative.

A general model for our data is that Rad54 and Rdh54 occupy unique binding sites with in the same Rad51 filaments which allows Rdh54 to slow the Rad54 mediated conversion of nascent D-loops into mature D-loops. The kinetic delay extends the lifetime of active Rad51 filaments allowing them to promote template switching, secondary strand invasion outcomes, or regulate the on-set of BIR (Fig 7A and 7B). If Rdh54 is persistently delayed, then it can regulate crossover/non-crossover outcomes. The key output of this mechanism is reduced LOH outcomes. The influence of this mechanism on internal strand invasions and may also be protective by reducing premature removal Rad51 allowing filaments to continue the homology search.

The biological consequences may all be related to the fact that Rad51 lacks the capacity to discriminate between ssDNA and dsDNA. Rad51 binding to dsDNA during HR is non-productive and can ultimately lead to chromosome mis-segregation [46]. Rdh54 is the major translocase involved in removing Rad51 from dsDNA keeping an active pool of enzyme that can bind to ssDNA increasing the likelihood of productive activity. Recently, Rdh54 has been shown to stabilize Rad51 on ssDNA against ATP depletion, and against the activity of the anti-recombinase Srs2 [49]. Stabilization of Rad51 on ssDNA is another mechanism to control the pool of productive Rad51. Our work extends these observations to stabilization of Rad51 at D-loop intermediates. Rad51 stabilization may help explain some of the previous phenotypes proposed for Rdh54 including ICTS, interhomolog recombination, and the partial redundancy with Rad54. In the case of ICTS, a secondary strand invasion event is required for D-loop movement [50]. This reinvasion event may only be possible if Rad51 is stabilized. Likewise, interhomolog recombination will occur infrequently when only one sister chromatid is cut, as this is the preferred substrate. Secondary invasion events may be necessary to switch chromosome templates to promote interhomolog gene conversion. Finally, the partial redundancy with *RAD54* may be explained by stabilization of Rad51 on ssDNA and at D-loops. The net result being that the secondary strand invasion activity promoted by Rdh54 takes on a primary role in strand invasion when Rad54 is absent. While stabilization of Rad51 may allow completion of HR in the absence of Rad54, HR occurs at significantly reduced efficiency consistent with Rad54’s role as a general catalyst. More recent work, including this study, imply that HR works most efficiently when the two Rad54 paralogs work together to regulate Rad51 dynamics [15].

We used a combination of genetic and biochemical experiments to show that the *S*. *cerevisiae* DNA motor protein Rdh54 acts as a kinetic regulator of D-loop maturation during HR. A two Rad54 paralog system is also conserved in most eukaryotes. For example, in higher eukaryotes Rdh54 is believed to be conserved as RAD54B [70], and Rad54 is conserved as RAD54L [47]. Currently, it remains unclear if RAD54B is a direct homolog of Rdh54 or Rad54. This leads to the possibility that RAD54B may only be functionally homologous to Rdh54. Along these lines, RAD54B shares several functional similarities to Rdh54, including roles in cell cycle recovery from DNA damage and removal of RAD51 from dsDNA [71–73]. Future work will be directed at understanding if the regulation of genetic exchange by Rdh54 is conserved in higher eukaryotes, how movement of Rdh54 relieves the barrier to mitotic crossover formation, and the precise mechanisms of Rdh54 activity during ICTS.

## Materials and methods

### Protein purification

Purification of Rad51, RPA, GST-Rad54, and GST-Rdh54 were carried out as previously described [48,74–76]. 6xHis-Rdh54 and 6xHis-Rdh54K318R were purified as follows. His_6_-Rdh54 in pET21b vector was expressed in *Escherichia coli* competent cells. Cells were grown in 6 liters of LB media or SB media at 37°C until they reached an OD_600_ of 0.4 to 0.6, then the temperature was shifted to 16°C and the cells were induced with 500 μM isopropyl β-D-1-thiogalactopyranoside (IPTG). After overnight expression, the cells were harvested and frozen at -80°C. After freezing for at least 30 minutes, cell pellets were re-suspended in 25 mL lysis buffer (25 mM Tris-Cl pH 7.5, 1000 mM NaCl, 10 mM Imidazole, 0.01% NP-40, 10% Glycerol, 5 mM BME, Protease inhibitor cocktails and 2 mM PMSF) per 3 liters of cell culture. Cells were sonicated 10 times with a 65% amplitude cycle for 15 seconds on and 45 seconds off. The lysates were clarified by ultracentrifugation for 45 minutes at 15,000 rpm. Clarified lysates were incubated with 2 mL settled volume of Ni-NTA Superflow Agarose for 1 hour at 4°C. The agarose was then washed by 10 column volumes (CV) of lysis buffer and 10 CV of wash buffer (25 mM Tris-Cl pH 7.5, 200 mM NaCl, 10 mM Imidazole, 0.01% NP-40, 10% Glycerol, 5 mM BME). After washing, the agarose was packed in a 10 mL poly-prep chromatography column and then drained. The protein was then eluted with 4 CV elution buffer (wash buffer + 190 mM Imidazole). Peak fractions were collected and then applied to a HiScreen SP FF column (Cytiva, Code No. 28950513, CV = 4.7 mL). The protein was bound to the column in buffer A (25 mM Tris-Cl pH 7.5, 0 mM KCl, 0.01% NP-40, 10% Glycerol, 5 mM BME, 1 mM EDTA). Then the column was washed by 3 CV buffer A. The protein was eluted by a gradient increasing buffer B (buffer A + 1000 mM KCl). The protein would be eluted at around 35% to 65% buffer B. Peak fractions were pooled and concentrated to 2 mL by a protein concentrator spin column at 3500 rpm. Then the protein was applied to a HiPrep Sephacryl S-300 HR column pre-equilibrated with SEC buffer (30 mM Na-HEPES pH 7.5, 400 mM NaCl, 10% Glycerol, 1 mM EDTA, 0.01% NP-40, 5 mM BME). The peak fractions were collected and concentrated to around 500 μL by a protein concentrator spin column (10 kDa MWCO) at 3500 rpm. The protein was then quantified by the absorbance at 280 nm. Samples were aliquoted and stored at -80°C.

### Yeast strain generation

Gene deletions were generated by PCR amplification of *KanMX* selectable marker with 50 nt of homology upstream and downstream of the ORF of the *RDH54* gene and transformed into WT-15D with selection on YPD+G418 (500 μg/ml). Genetic knockouts were confirmed by amplification of yeast genomic DNA with primers that annealed 250 bp upstream or downstream of the *RDH54* ORF. Subsequent deletions were generated by amplification of the region of the genome and transforming WT-11C. A similar strategy was used for *RAD54* deletions. For genetic knock-ins, a *KanMX* marker was integrated 125 bp downstream of the *RDH54* stop codon in a plasmid containing the *RDH54* gene. *WT-KanMX* and *rdh54K318R-KanMX* were PCR amplified and used to replace the endogenous *RDH54* locus in both WT-11C and WT-15D. Integration was confirmed by PCR. Full strain genotypes are in S4 Table. All diploids were made by crossing the parent haploid strains and selecting zygotes on YPD +noursethricin (67 μg/ml)+hygromycin B (200 μg/ml).

### Red/White homologous recombination assay

The WT strain used in this assay as well as the procedure for diploid formation are described here [53,55]. The genotypes for modifications to these strains can be found in S4 Table. The assay was performed by growing the appropriate strain overnight in YP +2% raffinose. The next day cells were diluted to an OD_600_ of 0.2 and allowed to reach an OD_600_ of 0.4–0.5 followed by the expression of I-SceI by the addition of 2% galactose. Cells were allowed to grow for an additional 1.5 hours after which they were plated on YP+2% dextrose and allowed to grow for 48 hours. After 48 hours they were placed in the 4°C overnight to allow further development of red color. The number of white, red, and sectored colonies was then counted followed by replica plating onto YPD+ hygromycin B (200 μg/ml) and YPD+ nourseothricin (67 μg/ml, clonNat) for analysis of recombination outcomes. Technical duplicate plates were also plated onto YNBR (+ade) +2% galactose to measure reinduction of I-Sce1, indicating the fraction of cleaved sister chromatids. Finally, strains were also replica plated on YNB (-URA/-MET) + 2% dextrose to insure proper chromosome segregation. The data were analyzed by counting colonies that were red, white, or sectored, and then counting colony survival on different antibiotic sensitivities. The data for each category was then divided by the total population of sectored colonies. The standard deviation between biological replicates analyzed for at least three independent experiments. The red and white colonies were analyzed in similar ways with the exception that the number of red colonies that were not cleaved by I-Sce1(as assayed by reinduction) were subtracted from the total red cell population, and only recombinants were used in the analysis.

### D-loop assay

D-loop formation experiments were performed in HR buffer (30 mM Tris-OAc [pH 7.5], 50 mM NaCl, 10 mM MgOAc_2_, 1 mM DTT, 0.2 mg/ml BSA) using an Atto647N labeled DNA duplex (15 nM) consisting of 21 nt overhang that was homologous to a region on the pUC19 plasmid. Rad51 (300 nM) was incubated with recipient DNA at 30°C for 15 minutes. The resulting Rad51 filaments were then mixed with indicated concentrations of Rad54, Rdh54 or both, RPA (500 nM) and pUC19 plasmid (0.3 nM). Reactions were quenched after indicated periods of time and treated with 1 unit of Proteinase K at 37°C for 20 minutes. The reactions were then resolved by electrophoresis on a 0.9% agarose gel and imaged for fluorescence using a Typhoon FLA 9500. The sequences of the oligos used in this study can be found in S4 Table.

### 3’ end extension assay

3’ end extension assays experiments where set-up as describe in the D-loop assays with the exception that dNTP’s (1 mM) and Klenow (exo-)(NEB) were added after 5 minutes of D-loop formation. Experiments were performed with either 21 nt of ssDNA homology or 65 nt of ssDNA homology to the pUC19 plasmid. The recipient DNA molecules were labeled by annealing a shorter non-homologous piece of DNA that was 5’ end labeled with Atto647N. The reactions were quenched as described above and incubated with 1 unit of Proteinase K for 37°C for 20 minutes. The reactions were then resolved by electrophoresis on a 0.9% agarose gel and imaged for fluorescence using a Typhoon FLA 9500. The sequences of the oligos used in this study can be found in S4 Table.

### ATP hydrolysis assay

ATP hydrolysis assays were performed in HR buffer (30 mM Tris–OAc [pH 7.5], 50 mM NaCl, 20 mM MgOAc, 1 mM DTT, 0.2 mg/ml BSA) in the presence of 1 mM ATP and trace amounts of γ^32^P–ATP. All reactions were performed at 30°C and contained pUC19 plasmid dsDNA (50 ng/μl). Aliquots were removed at specified time points and quenched with 25 mM EDTA and 1% SDS. The quenched reactions were spotted on TLC plates (Millipore, Cat. No. HX71732079) and resolved in 0.5 M LiCl plus 1 M formic acid. Dried TLC plates were exposed to phosphorimaging screen and scanned with a GE Healthcare Life Sciences Typhoon FLA 9500 biomolecular imaging system.

### Total protein measurement

Total protein measurements were made as described in Silva et al. [63]. Briefly, *RAD54-YFP* (strain ML1067) or *RDH54-YFP* (strain ML906) cells were estimated by measuring total nuclear YFP fluorescence intensities using Volocity software.

## Supporting information

S1 Table All red/white sectored outcomes CO/NCO/BIR. (PDF)

S2 Table All Solid red outcomes CO/NCO/BIR and BIR like. (PDF)

S3 Table All solid white outcomes CO/NCO/BIR and BIR like. (PDF)

S4 Table Yeast strains used in this study. (PDF)

S5 Table DNA oligonucleotides used in this study. (PDF)

S1 Fig RDH54 has different effects in Solid red or Solid white colonies. (A). Quantification of sectored red colonies in which both sister chromatid has undergone STGC for BIR or BIR like, CO, and NCO for *WT*, *RDH54/rdh54Δ*, *rdh54Δ/rdh54Δ*, and *rdh54K318R/rdh54K318R* strains. The columns represent the mean, and the error bars represent the standard deviation of at least 3 independent experiments. (B). Quantification of sectored white colonies in which both sister chromatid has undergone LTGC for BIR or BIR like, CO, and NCO for *WT*, *RDH54/rdh54Δ*, *rdh54Δ/rdh54Δ*, *and rdh54K318R/rdh54K318R* strains. The columns represent the mean, and the error bars represent the standard deviation of at least 3 independent experiments.
(TIF)

S2 Fig Rdh54 does not alter Rad54 ATPase functions at physiological ratios. **(A).** ATPase assay illustrating the fraction of Rad54 ATP hydrolysis in the presence and absence of Rad51 with and without Rdh54K318R. The bars represent the mean and standard deviation of three independent experiments. **(B).** Quantification of the number of fluorescently labeled Rad54 (N = 100) and Rdh54 (N = 100) molecules in yeast nucleus. The line and error bars represent the mean and 95% confidence interval of the data. The red number above the data is the mean molecules per cell.
(TIF)

S3 Fig N-terminal chimeras of Rad54 and Rdh54 show differences in D-loop turnover. **(A).** Schematic illustrating chimeric constructs for ^Rdh54N^Rad54, and ^Rad54N^Rdh54. These mutants alter the position of translocase binding on Rad51 filaments. **(B).** Representative agarose gel illustrating the formation and disruption of D-loops in the presence of Rad54 (left), ^Rdh54N^Rad54 (middle left), Rdh54 (middle right), ^Rad54N^Rdh54 (right). The recipient DNA used in these studies is 21 nt of homologous ssDNA and 36 bp of non-homologous dsDNA. **(C).** Quantification of D-loop formation and disruption in the presence of Rad54 (left), ^Rdh54N^Rad54 (middle left), Rdh54 (middle right), ^Rad54N^Rdh54 (right). The columns and error bars represent the mean and standard deviation of independent experiments.
(TIF)

S4 Fig Gene conversion outcomes are unaffected in N-terminal chimeric Rad54 and Rdh54. **(A)** Graph representing the red/white/sectored colony outcomes for all strains used in this paper. Strains are labeled in the figure. The bars and error bars represent the mean and standard deviation of at least three independent experiments.
(TIF)

S5 Fig Comparison of N-terminal Chimeras for solid red and solid white colonies. (A). Quantification of sectored red colonies in which both sister chromatids have undergone STGC for BIR or BIR like, CO, and NCO for *WT*, *rdh54Δ/rdh54Δ*, ^*rad54N*^*rdh54/* ^*rad54N*^*rdh54*, *and* ^*rdh54N*^*rad54/* ^*rdh54N*^*rad54* strains. The WT and *rdh54Δ* strains are reproduced from S1 Fig. The columns represent the mean, and the error bars represent the standard deviation of at least 3 independent experiments. (B). Quantification of sectored white colonies in which both sister chromatid has undergone LTGC for BIR or BIR like, CO, and NCO for *WT*, *rdh54Δ/rdh54Δ*, ^*rad54N*^*rdh54/* ^*rad54N*^*rdh54*, and ^*rdh54N*^*rad54/* ^*rdh54N*^*rad54* strains. The *WT* and *rdh54Δ* strains are reproduced from S1 Fig. The columns represent the mean, and the error bars represent the standard deviation of at least 3 independent experiments.
(TIF)

S6 Fig Frequency differences between WT and mutant *RDH54* strains with BIR and CO outcomes. (A). Bar graph representing the differences between *rdh54Δ/rdh54Δ*, ^*rad54N*^*Rdh54/* ^*rad54N*^*Rdh54*, and ^*rdh54N*^*Rad54/* ^*rdh54N*^*Rad54* for BIR outcomes in Solid White, Solid Red, and Sectored outcomes. (B). Bar graph representing the differences between *rdh54Δ/rdh54Δ*, *rdh54K318R/rdh54K318R*, ^*rad54N*^*Rdh54/* ^*rad54N*^*Rdh54*, and ^*rdh54N*^*Rad54/* ^*rdh54N*^*Rad54* for CO outcomes in Solid White, Solid Red, and Sectored outcomes. The data was generated by dividing the appropriate mutant strain by the WT.
(TIF)

S1 Data Supplementary data. (XLSX)
