# Supplementary material for: Rdh54 stabilizes Rad51 at displacement loop intermediates to regulate genetic exchange between chromosomes
Source: PLoS Genet. 2022 Sep 13;18(9):e1010412. doi: 10.1371/journal.pgen.1010412 (PMC9506641; doi:10.1371/journal.pgen.1010412)
Supplement: S1 Table — (PDF) [file pgen.1010412.s001.pdf]

Supplemental Table S1
All red/white sectored outcomes CO/NCO/BIR

Strain	Red	White	Sectored	Total	CO	NCO	BIR	Chromosome loss
WT	457	194	960	1611	398	504	58	1/145
WT-KanMX/ WT-KanMX	307	50	548	905	200	323	25	0/325
RDH54/rdh54Δ	233	52	339	624	140	163	36	0/223
rdh54Δ/rdh54Δ	238	97	301	636	104	145	52	0/67
rad54Δ/rad54Δ	410	1	0	411	N/A	N/A	N/A	N/A
rdh54K318R/ rdh54K318R	285	21	381	687	56	311	14	0/381
^{rdh54N}*Rad54/* ^{rdh54N}*Rad54*	568	56	936	1560	286	626	51	0/324
^{rad54N}*Rdh54/* ^{rad54N}*Rdh54*	453	60	632	1145	166	381	85	0/79
