# Supplementary material for: Rdh54 stabilizes Rad51 at displacement loop intermediates to regulate genetic exchange between chromosomes
Source: PLoS Genet. 2022 Sep 13;18(9):e1010412. doi: 10.1371/journal.pgen.1010412 (PMC9506641; doi:10.1371/journal.pgen.1010412)
Supplement: S2 Table — (PDF) [file pgen.1010412.s002.pdf]

Supplemental Table S2
All solid red outcomes CO/NCO/BIR and BIR like

	CO	NCO	BIR or BIR like	Total	Number Uncut
**WT**	**9**	**139**	**4**	**158**	**19**
RDH54/rdh54Δ	**6**	**95**	**6**	**111**	**16**
rdh54Δ/rdh54Δ	**12**	**259**	**20**	**289**	**30**
rad54Δ/rad54Δ	**0**	**0**	**0**	**379**	**378**
rdh54K318R/ rdh54K318R	**4**	**216**	**4**	**251**	**35**
^{rdh54N}***Rad54/*** ^{rdh54N}***Rad54***	**4**	**49**	**2**	**55**	**4**
^{rad54N}***Rdh54/*** ^{rad54N}***Rdh54***	**18**	**238**	**15**	**271**	**26**
