# Supplementary material for: Rdh54 stabilizes Rad51 at displacement loop intermediates to regulate genetic exchange between chromosomes
Source: PLoS Genet. 2022 Sep 13;18(9):e1010412. doi: 10.1371/journal.pgen.1010412 (PMC9506641; doi:10.1371/journal.pgen.1010412)
Supplement: S4 Table — (PDF) [file pgen.1010412.s004.pdf]

Supplemental Table S4
Yeast Strains used in this study

Strains	Genotype*	Source or reference
LSY-2202-15D (WT)	*MAT***a** *ade2-n his3:NatMX4 met22:klURA3*	(Mazon et al. 2010)
LSY-2205-11C (WT)	*MAT*alpha *ade2-I lys2:GAL-ISCEI his3:HphMX4*	(Mazon et al. 2010)
JBC001 (15D)	*rdh54Δ:KanMX*	This study
JBC002 (11C)	*rdh54Δ:KanMX*	This study
JBC007 (11C)	*rad54Δ:KanMX*	This study
JBC010 (15D)	*rad54Δ:KanMX*	This study
JBC0011 (15D)	*RDH54:RDH54-KanMX*	This study
JBC0013 (11C)	*RDH54:RDH54-KanMX*	This study
JBC0014 (15D)	*RDH54:rdh54K318R-KanMX*	This study
JBC0016 (11C)	*RDH54:rdh54K318R-KanMX*	This study
JBC0039 (15D)	*RDH54:*^{rdh54N}*RAD54-KanMX*	This study
JBC0041 (11C)	*RDH54:*^{rdh54N}*RAD54-KanMX*	This study
JBC0043 (15D)	*RDH54:*^{rad54N}*RDH54-KanMX*	This study
JBC0045 (11C)	*RDH54:*^{rad54N}*RDH54-KanMX*	This study
Diploid (JBC001 X JBC002)	*rdh54:KanMX/rdh54:KanMX*	This study
Diploid (LSY-2202 X JBC001)	*RDH54/rdh54:KanMX*	This study
Diploid (JBC007 XJBC010)	*rad54Δ:KanMX/ rad54Δ:KanMX*	This study
Diploid (JBC0011 X JBC0013)	*RDH54:RDH54-KanMX/ RDH54:RDH54-KanMX*	This study
Diploid (JBC 0014 X JBC0016)	*RDH54:rdh54K318R-KanMX/ RDH54:rdh54K318R-KanMX*	This study
Diploid (JBC0039 X JBC0041)	*RDH54:*^{rdh54N}*RAD54-KanMX/ RDH54:*^{rdh54N}*RAD54-KanMX*	This study
Diploid (JBC0043 X JBC0045)	*RDH54:*^{rad54N}*RDH54-KanMX/ RDH54:*^{rad54N}*RDH54-KanMX*	This study

Reference

1. Mazón G, Symington Lorraine S. Mph1 and Mus81-Mms4 Prevent Aberrant Processing of Mitotic Recombination Intermediates. Molecular Cell. 2013;52(1):63-74. doi: https://doi.org/10.1016/j.molcel.2013.09.007.
