# Supplementary material for: Rdh54 stabilizes Rad51 at displacement loop intermediates to regulate genetic exchange between chromosomes
Source: PLoS Genet. 2022 Sep 13;18(9):e1010412. doi: 10.1371/journal.pgen.1010412 (PMC9506641; doi:10.1371/journal.pgen.1010412)
Supplement: S5 Table — (PDF) [file pgen.1010412.s005.pdf]

Supplemental Table S5
DNA oligonucleotides used in this study

DNA Name	Sequence
21-mer homolog	5′–AAT TCT CAT TTT ACT TAC CGG ACG CTA TTA GCA GTG GGT GAG CAA AAA CAG GAA GGC–3
21-mer label	5′–Atto647N–CAC TGC TAA TAG CGT CCG GTA AGT AAA ATG AGA ATT–3′
65-mer homolog	5’-AGGGAATGCCCGTTCTGCGAGGCGGTGGATCTCAACAGCGGTAAGATCCTTGAGAGTTTTCGCCCCGAAGAACGTTTTCCAATGATGAGC- 3’
65-mer label	5’-Atto647N-CCGCCTCGCAGAACGGGCATTCCCT
