# Supplementary figures and images for: Rdh54 stabilizes Rad51 at displacement loop intermediates to regulate genetic exchange between chromosomes
Source: PLoS Genet. 2022 Sep 13;18(9):e1010412. doi: 10.1371/journal.pgen.1010412 (PMC9506641; doi:10.1371/journal.pgen.1010412)

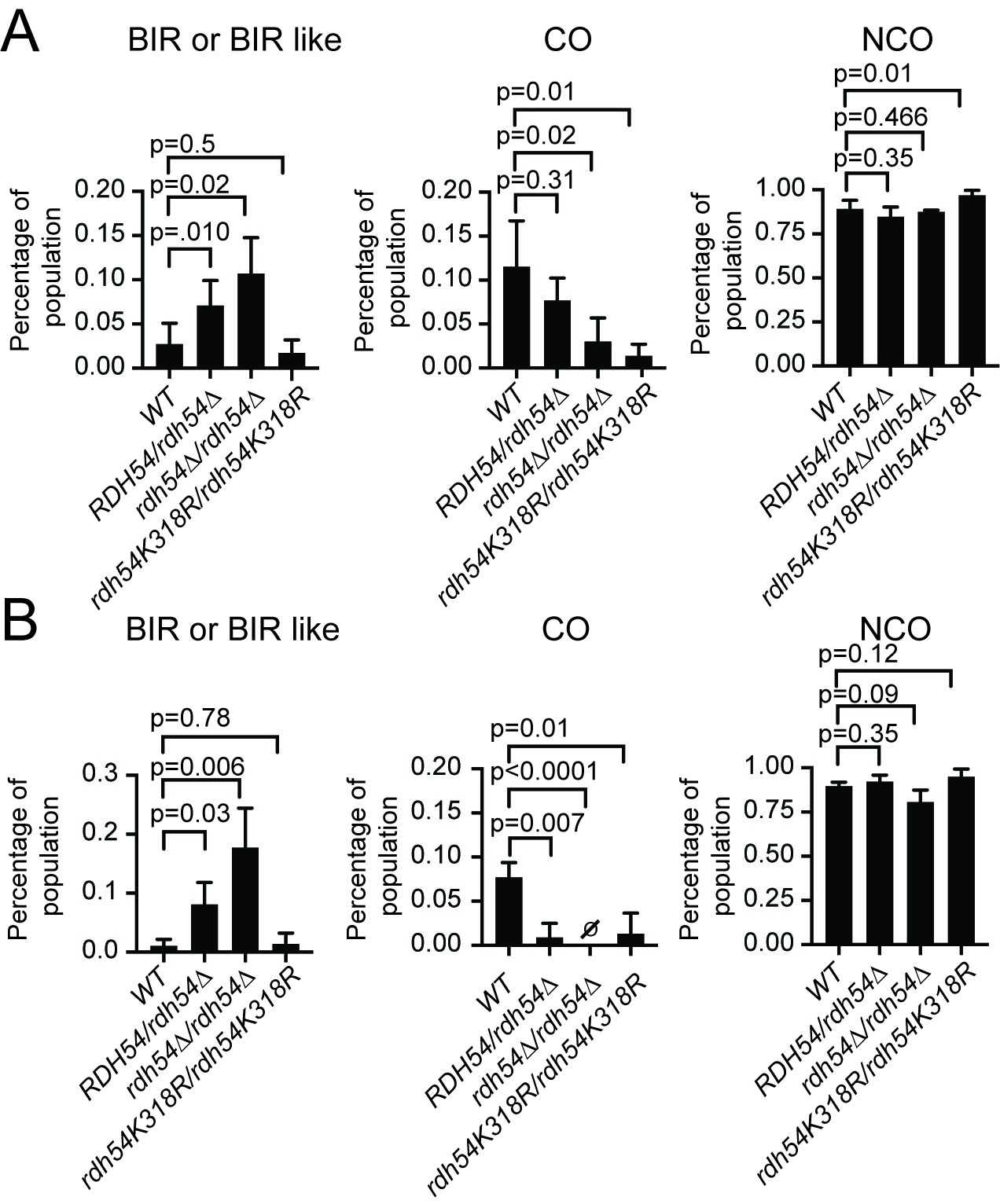

Supplement: S1 Fig — (A). Quantification of sectored red colonies in which both sister chromatid has undergone STGC for BIR or BIR like, CO, and NCO for WT, RDH54/rdh54Δ, rdh54Δ/rdh54Δ, and rdh54K318R/rdh54K318R strains. The columns represent the mean, and the error bars represent the standard deviation of at least 3 independent experiments. (B). Quantification of sectored white colonies in which both sister chromatid has undergone LTGC for BIR or BIR like, CO, and NCO for WT, RDH54/rdh54Δ, rdh54Δ/rdh54Δ, and rdh54K318R/rdh54K318R strains. The columns represent the mean, and the error bars represent the standard deviation of at least 3 independent experiments. (TIF) [file pgen.1010412.s006.tif]

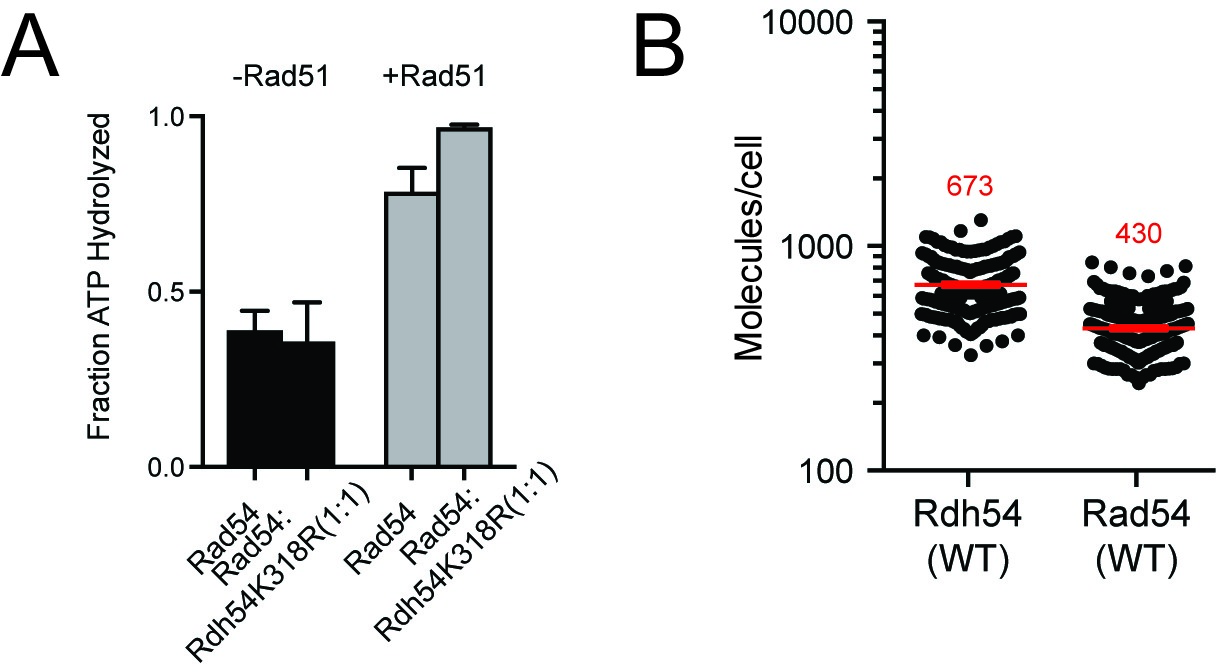

Supplement: S2 Fig — (A). ATPase assay illustrating the fraction of Rad54 ATP hydrolysis in the presence and absence of Rad51 with and without Rdh54K318R. The bars represent the mean and standard deviation of three independent experiments. (B). Quantification of the number of fluorescently labeled Rad54 (N = 100) and Rdh54 (N = 100) molecules in yeast nucleus. The line and error bars represent the mean and 95% confidence interval of the data. The red number above the data is the mean molecules per cell. (TIF) [file pgen.1010412.s007.tif]

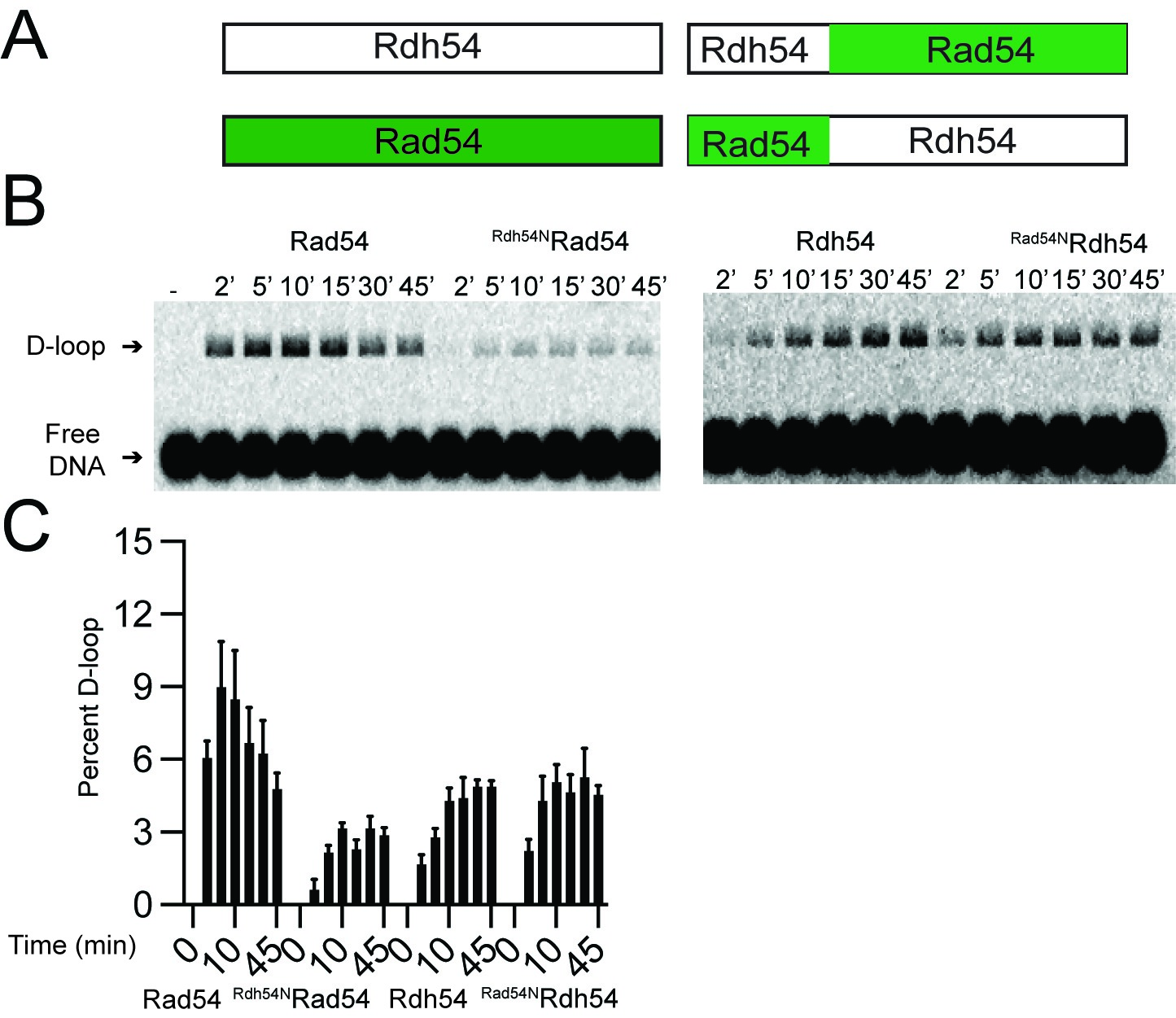

Supplement: S3 Fig — (A). Schematic illustrating chimeric constructs for Rdh54NRad54, and Rad54NRdh54. These mutants alter the position of translocase binding on Rad51 filaments. (B). Representative agarose gel illustrating the formation and disruption of D-loops in the presence of Rad54 (left), Rdh54NRad54 (middle left), Rdh54 (middle right), Rad54NRdh54 (right). The recipient DNA used in these studies is 21 nt of homologous ssDNA and 36 bp of non-homologous dsDNA. (C). Quantification of D-loop formation and disruption in the presence of Rad54 (left), Rdh54NRad54 (middle left), Rdh54 (middle right), Rad54NRdh54 (right). The columns and error bars represent the mean and standard deviation of independent experiments. (TIF) [file pgen.1010412.s008.tif]

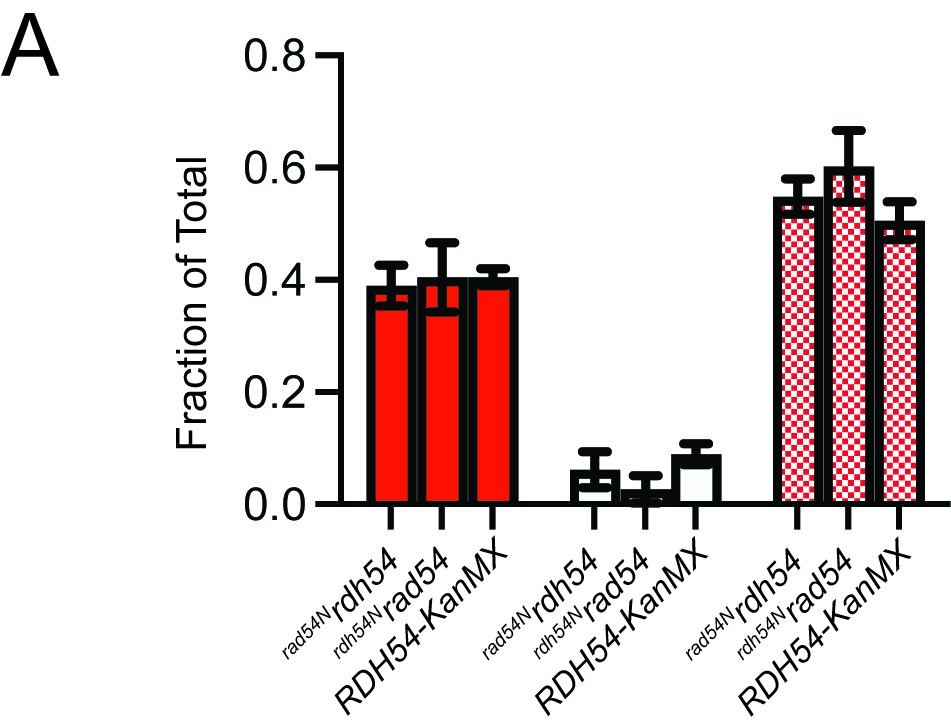

Supplement: S4 Fig — (A) Graph representing the red/white/sectored colony outcomes for all strains used in this paper. Strains are labeled in the figure. The bars and error bars represent the mean and standard deviation of at least three independent experiments. (TIF) [file pgen.1010412.s009.tif]

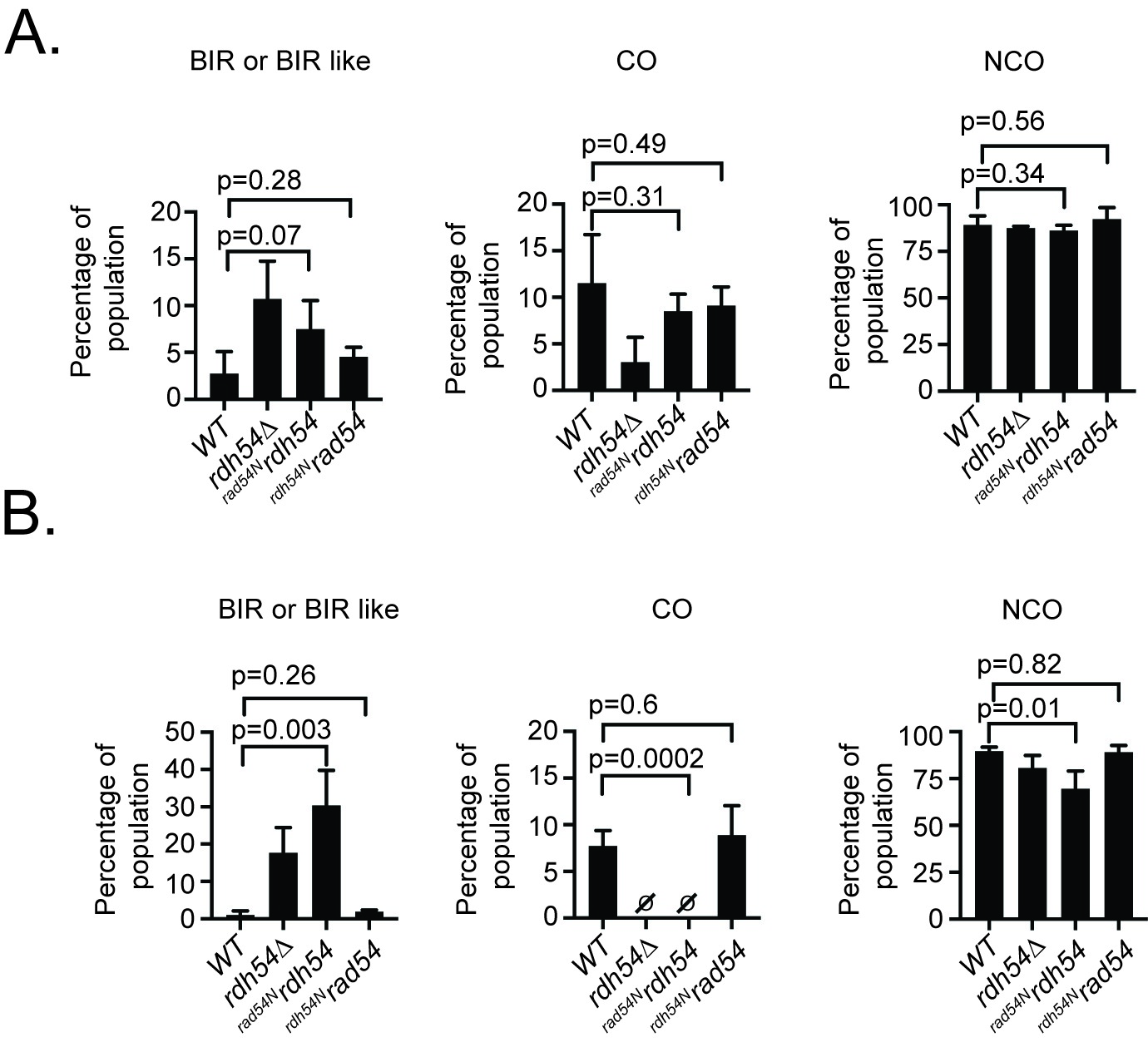

Supplement: S5 Fig — (A). Quantification of sectored red colonies in which both sister chromatids have undergone STGC for BIR or BIR like, CO, and NCO for WT, rdh54Δ/rdh54Δ, rad54Nrdh54/ rad54Nrdh54, and rdh54Nrad54/ rdh54Nrad54 strains. The WT and rdh54Δ strains are reproduced from S1 Fig. The columns represent the mean, and the error bars represent the standard deviation of at least 3 independent experiments. (B). Quantification of sectored white colonies in which both sister chromatid has undergone LTGC for BIR or BIR like, CO, and NCO for WT, rdh54Δ/rdh54Δ, rad54Nrdh54/ rad54Nrdh54, and rdh54Nrad54/ rdh54Nrad54 strains. The WT and rdh54Δ strains are reproduced from S1 Fig. The columns represent the mean, and the error bars represent the standard deviation of at least 3 independent experiments. (TIF) [file pgen.1010412.s010.tif]

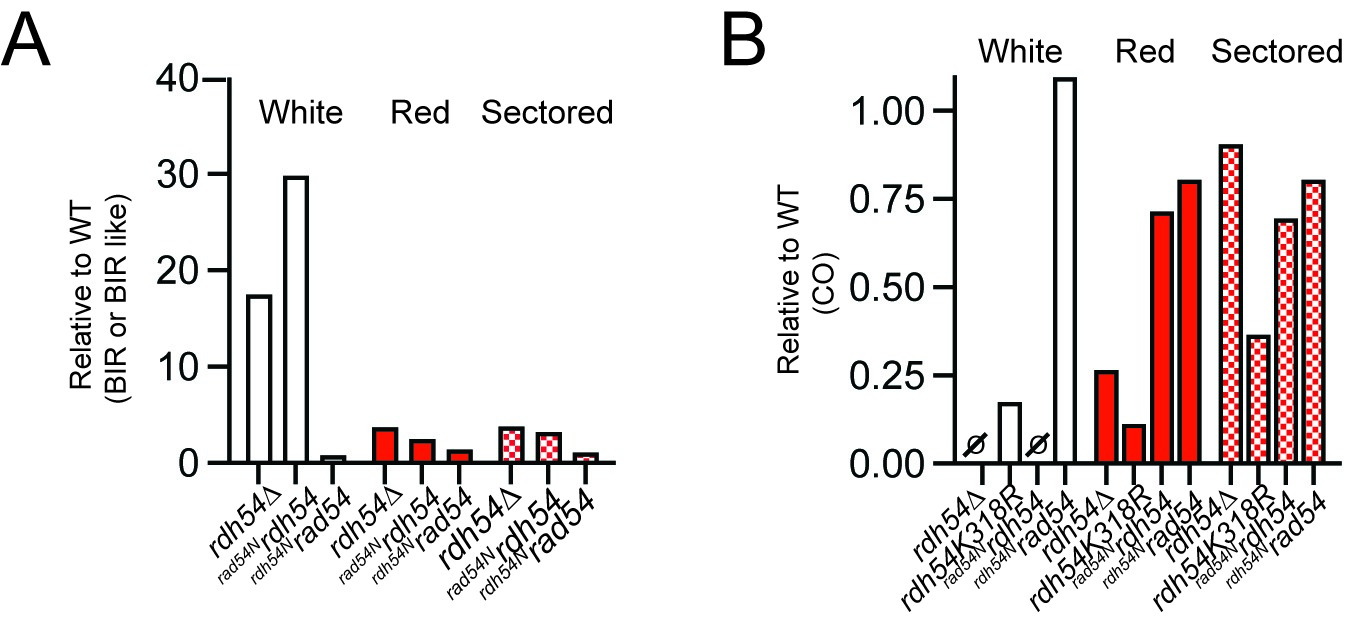

Supplement: S6 Fig — (A). Bar graph representing the differences between rdh54Δ/rdh54Δ, rad54NRdh54/ rad54NRdh54, and rdh54NRad54/ rdh54NRad54 for BIR outcomes in Solid White, Solid Red, and Sectored outcomes. (B). Bar graph representing the differences between rdh54Δ/rdh54Δ, rdh54K318R/rdh54K318R, rad54NRdh54/ rad54NRdh54, and rdh54NRad54/ rdh54NRad54 for CO outcomes in Solid White, Solid Red, and Sectored outcomes. The data was generated by dividing the appropriate mutant strain by the WT. (TIF) [file pgen.1010412.s011.tif]
